# Supplementary material for: Inhalable cardiac targeting peptide modified nanomedicine prevents pressure overload heart failure in male mice
Source: Nat Commun. 2024 Jul 18;15:6058. doi: 10.1038/s41467-024-50312-1 (PMC11258261; doi:10.1038/s41467-024-50312-1)
Supplement: Supplementary file 1 — Supplementary Information [file 41467_2024_50312_MOESM1_ESM.pdf]

Supplementary Materials

Inhalable cardiac targeting peptide modified nanomedicine prevents pressure overload heart failure in male mice

Haobo Weng[1,2,3#], Weijuan Zou[2,4#], Fangyan Tian[1,3,5#], Huilin Xie[1,3], Ao Liu[1,3], Wen Liu[1], Yu Liu[1], Nianwei Zhou[1], Xiaojun Cai[2,4], Jianrong Wu[2,4]*, Yuanyi Zheng[2,4]*, Xianhong Shu[1,3,6]*

1. Department of Echocardiography, Shanghai Institute of Medical Imaging, Zhongshan Hospital, Fudan University, Shanghai, P. R. China.

2. Shanghai Key Laboratory of Neuro-Ultrasound for Diagnosis and Treatment, Shanghai Sixth People's Hospital Affiliated to Shanghai Jiao Tong University School of Medicine, Shanghai, P. R. China.

3. Department of Cardiology, Shanghai Institute of Cardiovascular Disease, Zhongshan Hospital, Fudan University, Shanghai, P. R. China.

4. Department of Ultrasound in Medicine, Shanghai Institute of Ultrasound in Medicine, Shanghai Sixth People's Hospital Affiliated to Shanghai Jiao Tong University School of Medicine, Shanghai, P. R. China.

5. Department of Ultrasound Medicine, The Affiliated Hospital of Guizhou Medical University, Guiyang, China

6. Department of Ultrasound in Medicine, Shanghai Xuhui District Central Hospital, Shanghai, P. R. China.

# These authors contributed equally to this work.

* Corresponding author: wujianrong028@shsmu.edu.cn; zhengyuanyi@sjtu.edu.cn; shu.xianhong@zs-hospital.sh.cn

Supplementary Figures

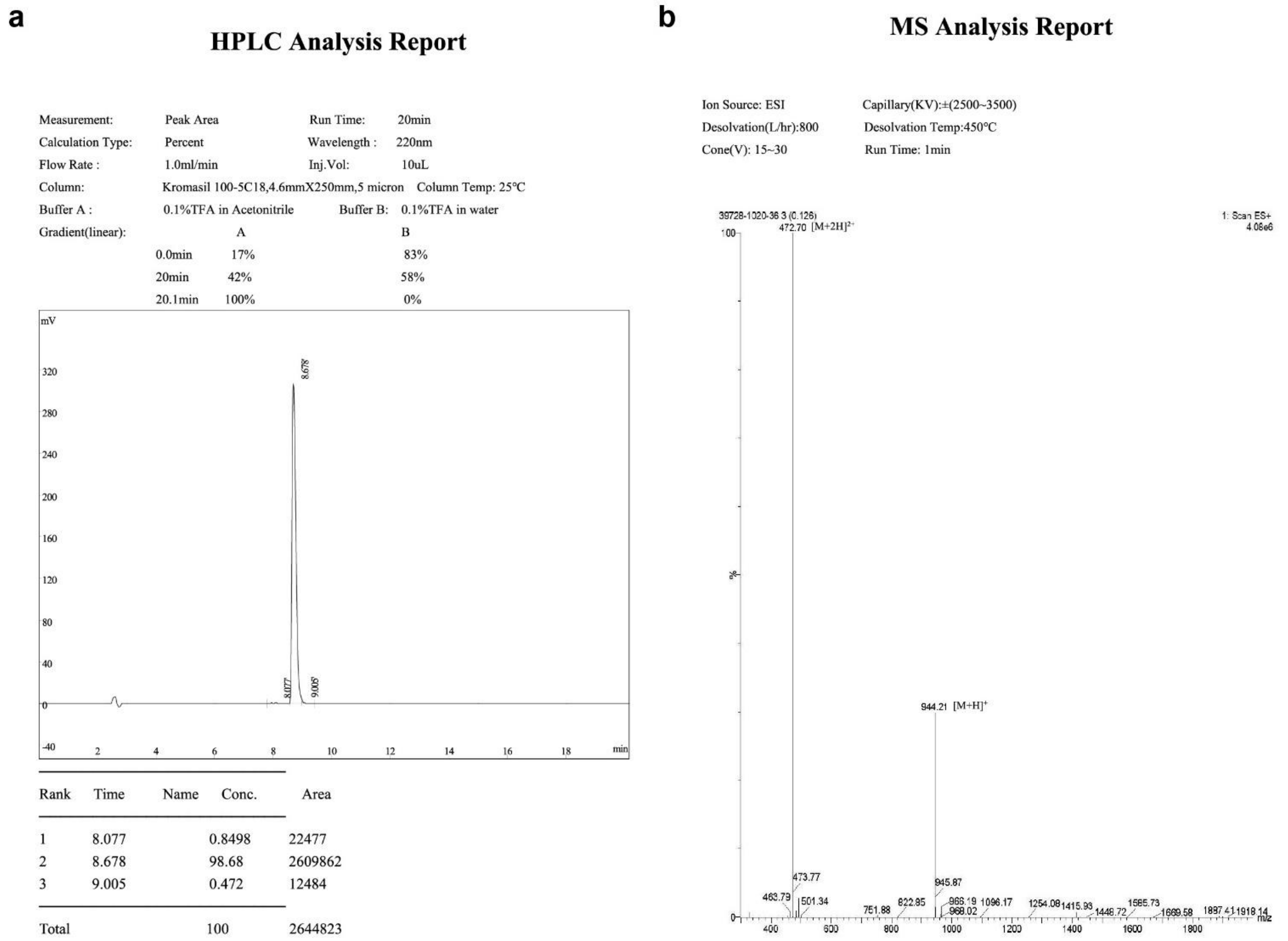

Supplementary Fig. 1. a, HPLC analysis and b, MS analysis reports of CTP.

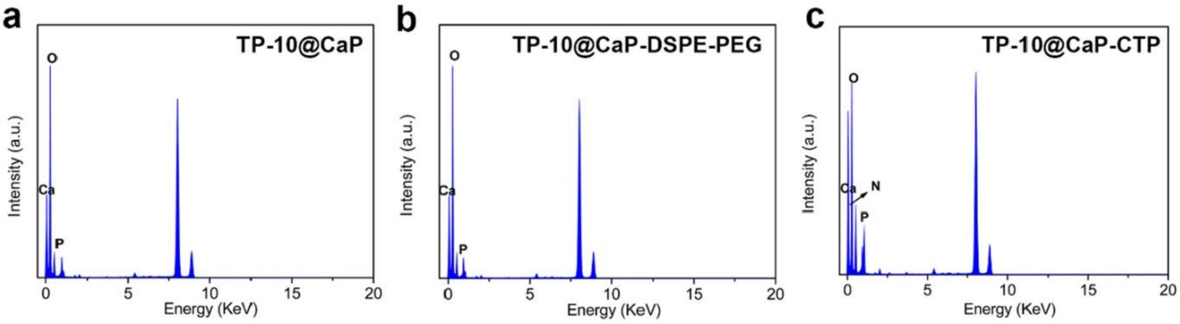

Supplementary Fig. 2. EDX patterns of a, TP-10@CaP, b, TP-10@CaP-DSPE-PEG and c, TP-10@CaP-CTP. One of three repetitions with similar results is shown here. Source data are provided as a Source Data file.

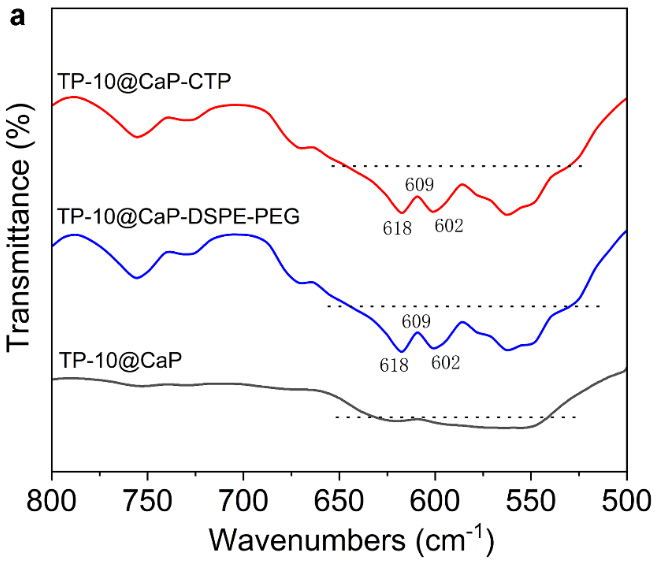

b

CaP-based materials	Splitting factor
TP-10@CaP	No calculable
TP-10@CaP-DSPE-PEG	3.15
TP-10@CaP-CTP	3.27

Supplementary Fig. 3. (a) FT-IR spectra of different prepared nanoparticles (enlarged image of Fig. 2f). (b) Splitting factors of different prepared nanoparticles was calculated by the sum of the heights of the valleys at 602 and 618 cm^{-1} and divided by the height of the peak between them at ~609 cm^{-1}. All heights were measured above a baseline drawn from approximately 645-530 cm^{-1}). One of three repetitions with similar results is shown here. Source data are provided as a Source Data file.

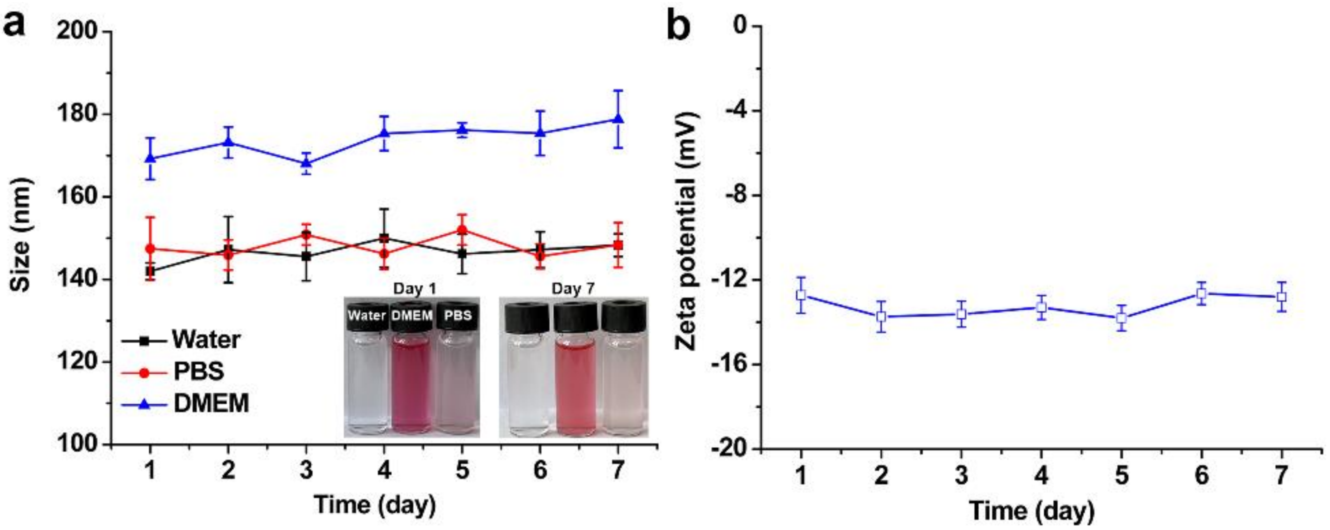

Supplementary Fig. 4. In vitro stability of nanoparticles. a, the hydrodynamic size of TP-10@CaP-CTP (0.4 mg/mL) dispersed in different mediums (water, PBS, and DMEM) for 7 days (n = 3 independent samples in each group). b, Zeta potentials of TP-10@CaP-CTP dispersions in PBS (pH 7.4) for 7 days (n = 5 samples independent samples in each group). Source data are provided as a Source Data file.

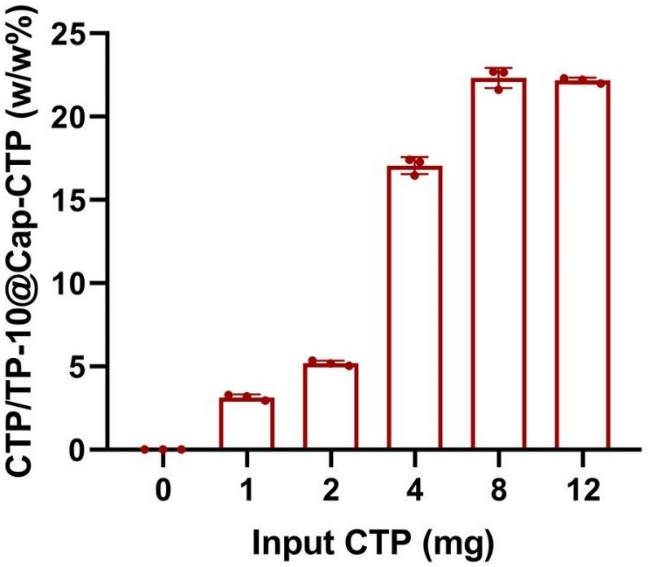

Supplementary Fig. 5. The CTP bonding amount and encapsulation efficiency after mixing of TP-10@CaP-DSPE-PEG (15 mg) and different amount of CTP (n = 3 independent samples in each group). Source data are provided as a Source Data file.

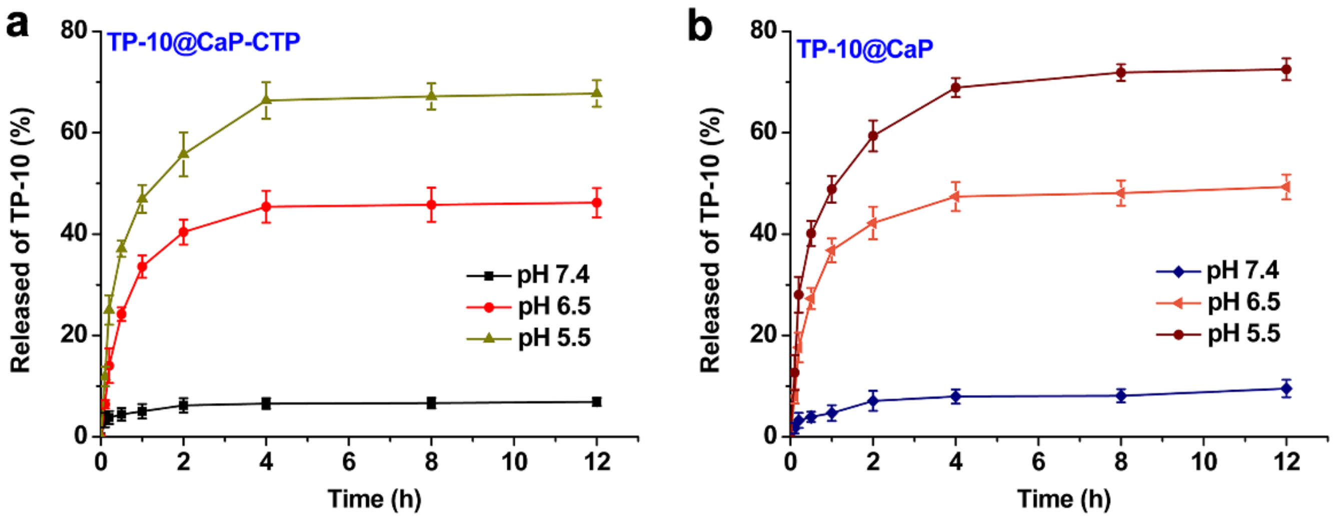

Supplementary Fig. 6. The release profiles of TP-10 from a, TP-10@CaP-CTP and b, TP-10@CaP at different pHs (n = 3 independent samples in each group). Source data are provided as a Source Data file.

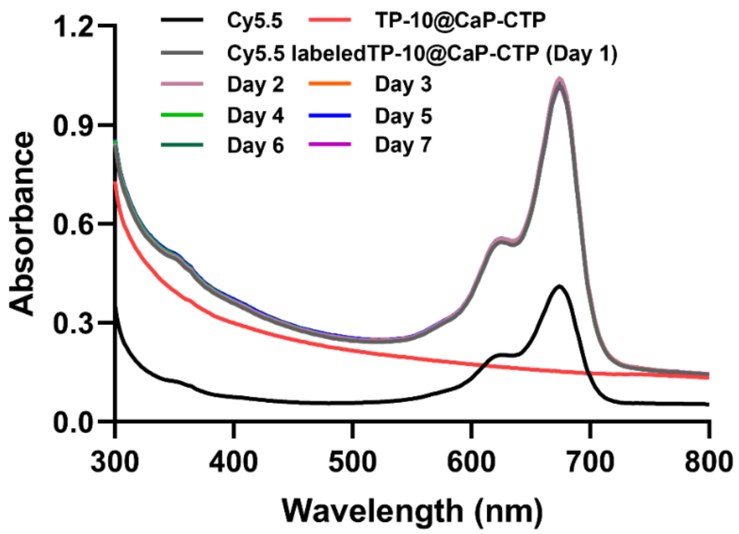

Supplementary Fig. 7. The UV-vis absorption spectra of TP-10@CaP-CTP, free Cy5.5 and Cy5.5-labelled TP-10@CaP-CTP dispersed in PBS for 7 days. Experiments were repeated three times independently. Source data are provided as a Source Data file.

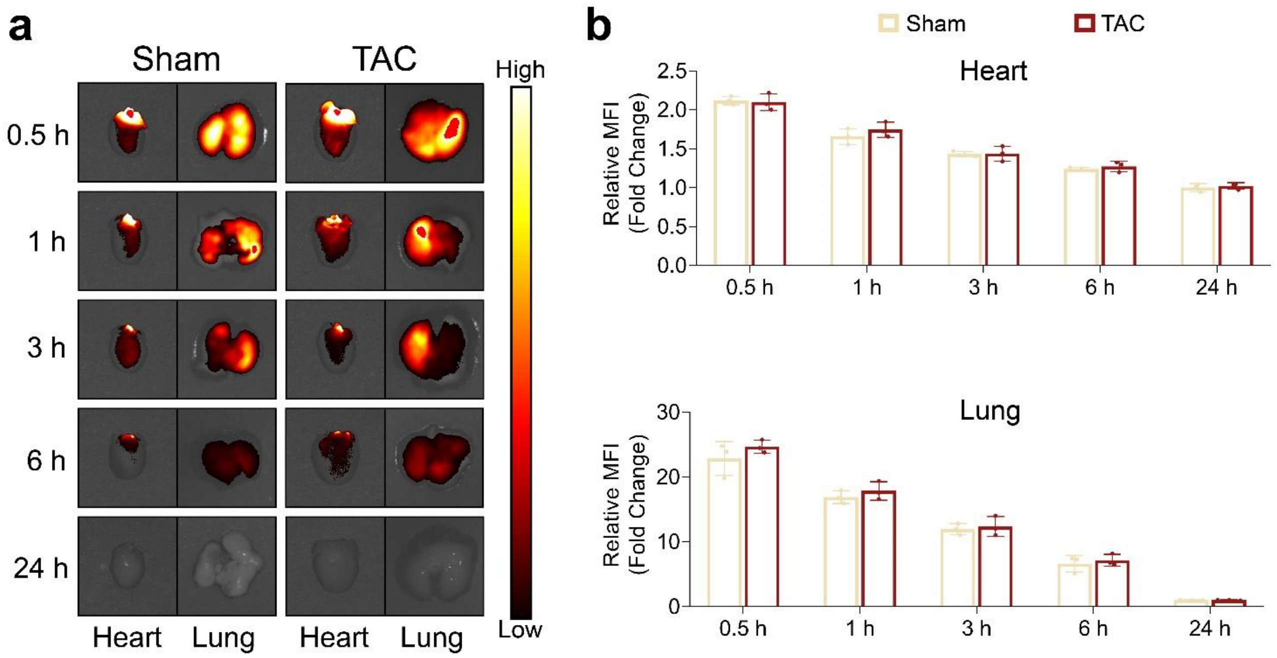

Supplementary Fig. 8. (a) Time-course fluorescence images and (b) quantification of fluorescence signal of free Cy5.5 in the lung and heart in the Sham- or TAC-operated mice. (n=3 hearts or lungs in each group). Source data are provided as a Source Data file.

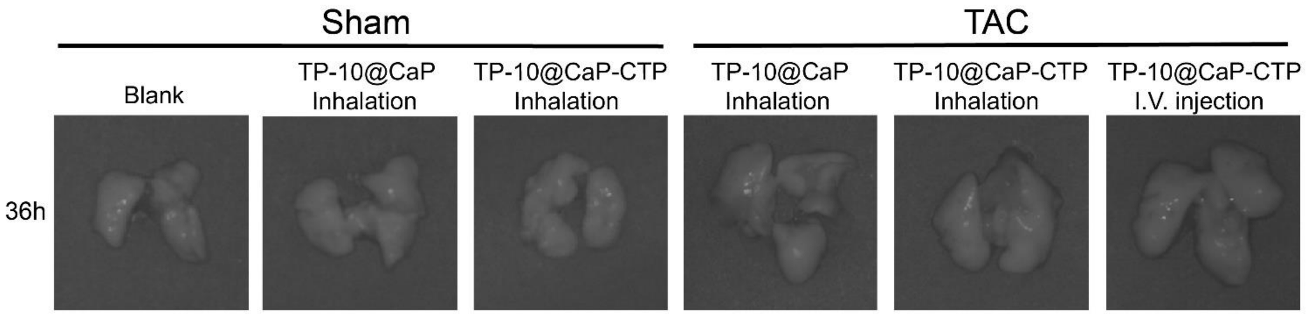

Supplementary Fig. 9. Ex vivo fluorescence images of lung tissue from mice received Cy5.5-labelled TP-10@CaP or TP-10@CaP-CTP treatment through inhalation approach or I.V. injection in TAC and Sham mice at 36 h. n = 3 lungs in each group.

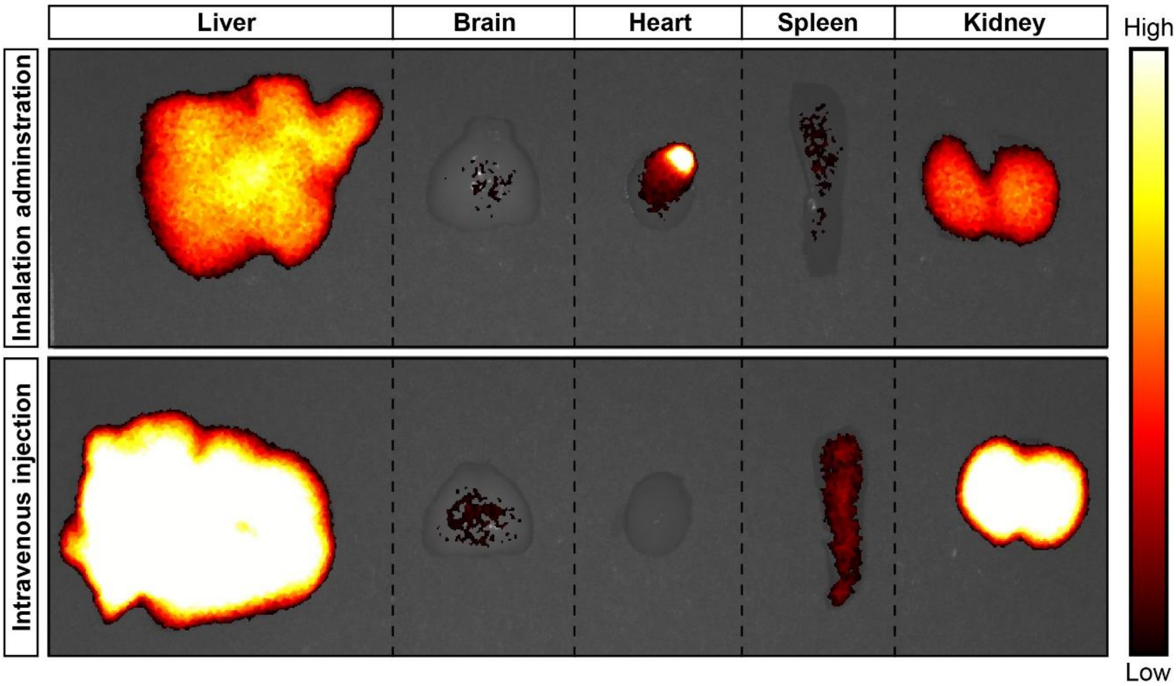

Supplementary Fig. 10. Distribution of Cy5.5-labelled TP-10@CaP-CTP nanoparticles in major organs (liver, brain, heart, spleen, and kidney) of the mice treated with Cy5.5-labelled TP-10@CaP-CTP (at 1 h) through inhalation administration or intravenous injection. n= 3 mice in each group.

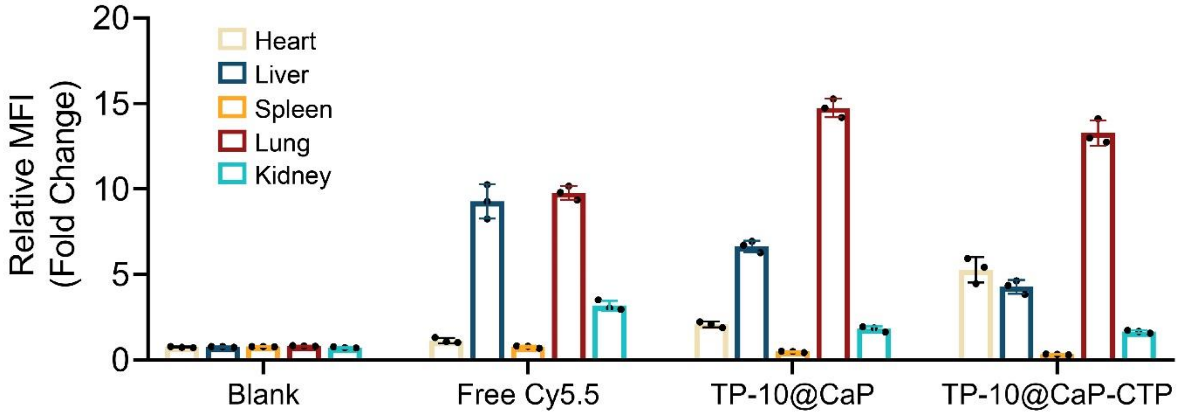

Supplementary Fig. 11. Quantitative assessment of Cy5.5 fluorescence intensities of various organs from mice after inhalation treatment with Cy5.5-labelled TP-10@CaP-CTP, TP-10@CaP and free Cy5.5. Tissues were harvested at 1 h after inhalation treatment and subjected to ex vivo fluorescent imaging detection (n=3 mice in each group). Source data are provided as a Source Data file.

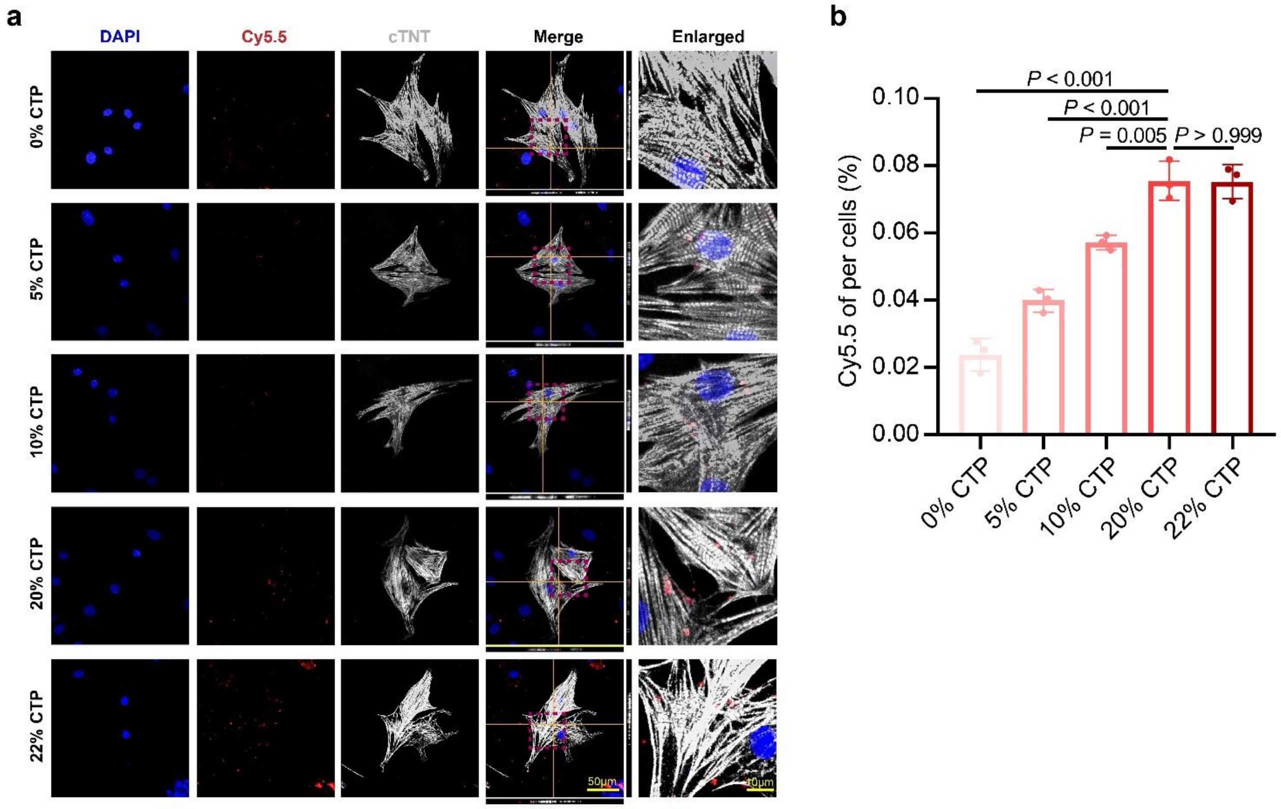

Supplementary Fig. 12. a, In vitro determination of targeting capacity of TP-10@CaP-CTP. Representative images of the intracellular uptake of Cy5.5-labelled TP-10@CaP-CTP with different amounts of CTP (0%, 5%, 10%, 20%, 22%, w/w%) in PE induced (pretreated with 100 μM PE for 24 h) neonatal rat ventricular myocytes (NRVMs). Scale bar, 50 μm. b, Quantitative assessment of fluorescence intensity from NRVMs. n = 3 biologically independent samples in each group. Statistical analysis was performed using one-way ANOVA with the Bonferroni multiple comparison correction. Source data are provided as a Source Data file.

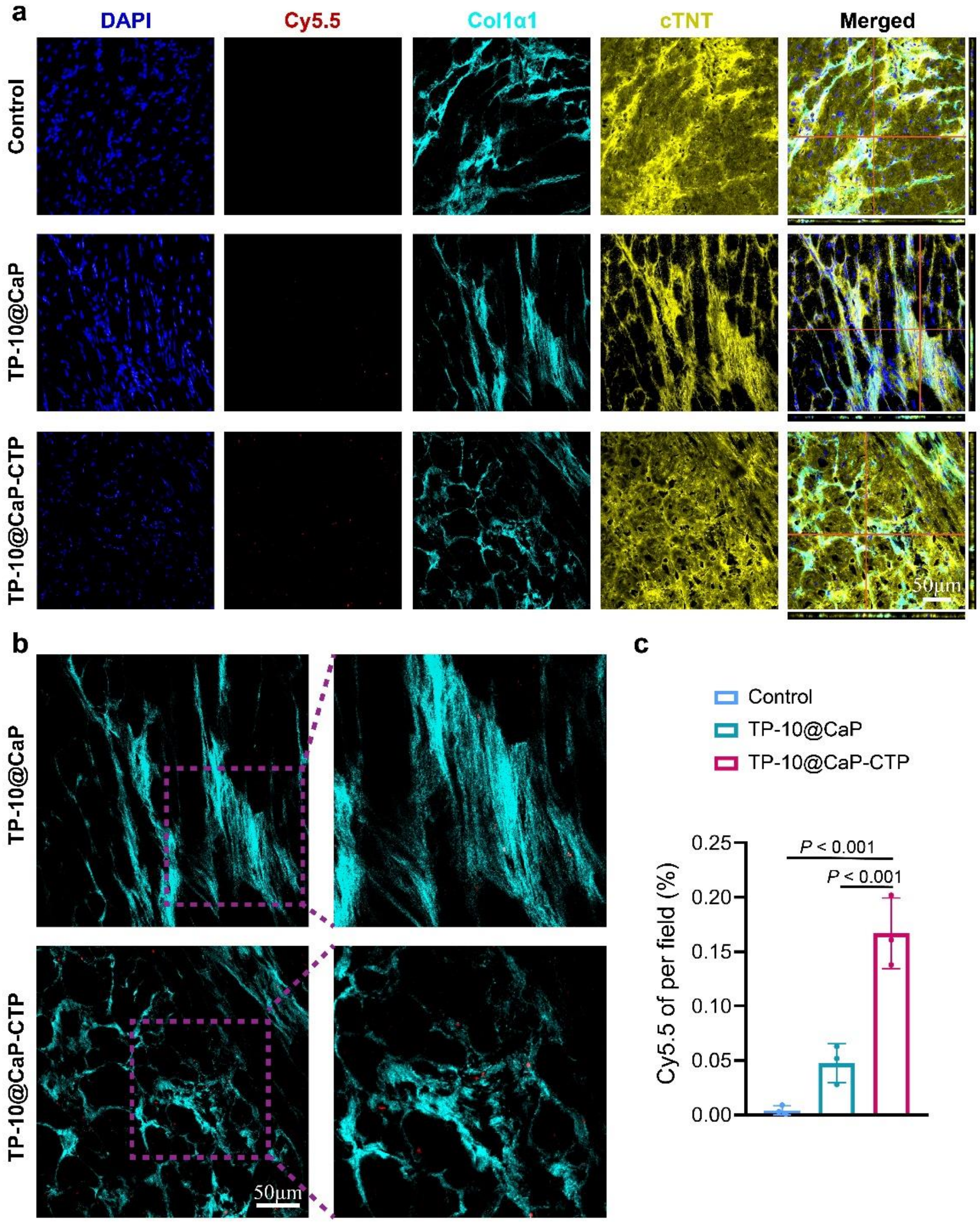

Supplementary Fig. 13. The accumulation of Cy5.5-labelled TP-10@CaP or TP-10@CaP-CTP nanoparticles in the fibrotic area of heart. a, Representative images of nanoparticles accumulation in the fibrotic area of heart after treated with PBS (blank control), Cy5.5-labelled TP-10@CaP and Cy5.5-labelled TP-10@CaP-CTP via inhalation. Scale bar, 50 μm. b, enlarged images of the distribution of Cy5.5-labelled TP-10@CaP and TP-10@CaP-CTP in the fibrotic area of heart. Scale bar, 50 μm. c, Quantitative assessment of Cy 5.5 fluorescence signals in the fibrotic areas. n = 3 biologically independent samples in each group. The results are presented as the mean ± SD. Statistical analysis was performed using one-way ANOVA with the Bonferroni multiple comparison correction. Source data are provided as a Source Data file.

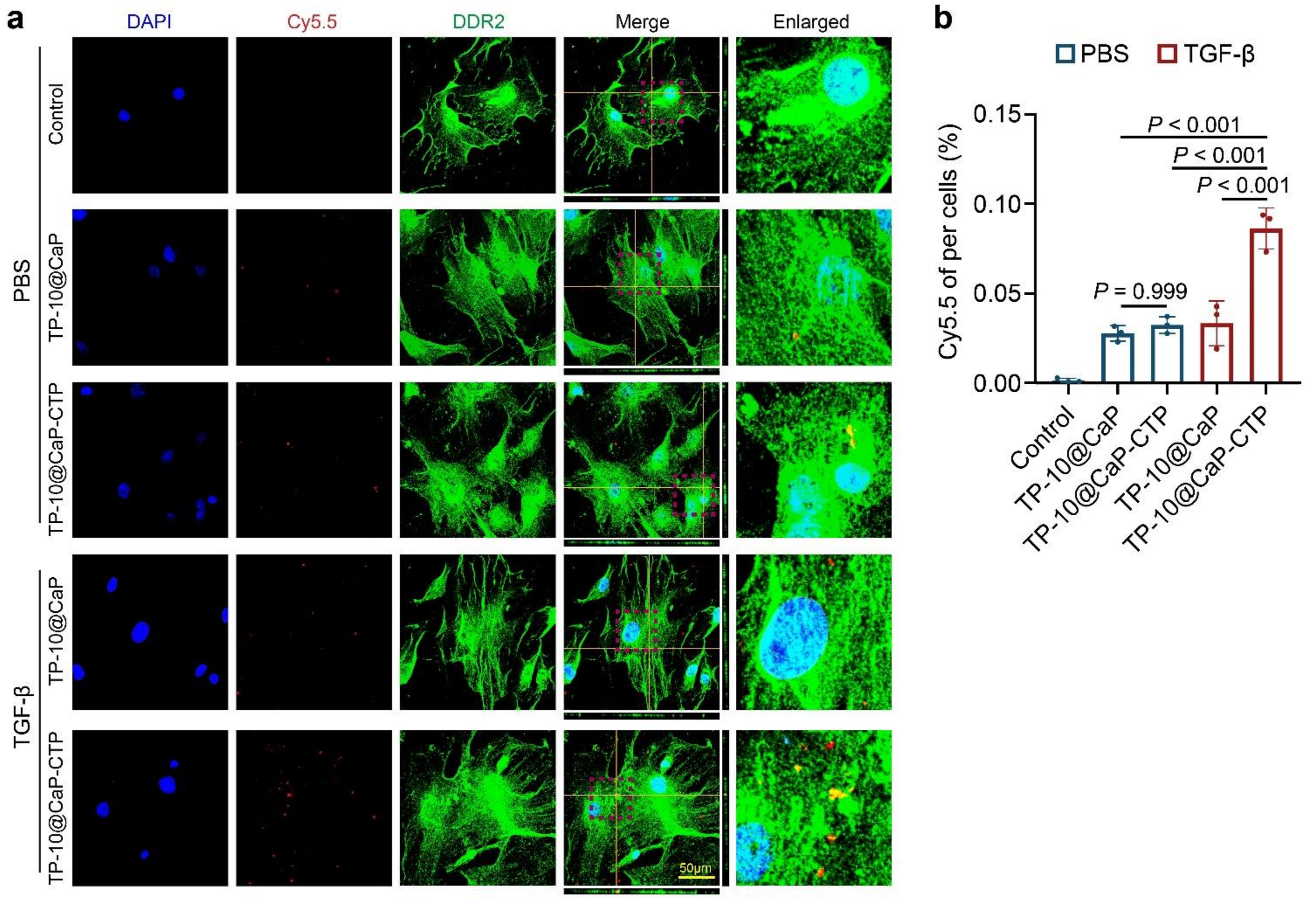

Supplementary Fig. 14. In vitro determination of targeting capacity of TP-10@CaP and TP-10@CaP-CTP in AMCFs. a, Representative images of the intracellular uptake of Cy5.5-labelled TP-10@CaP and TP-10@CaP-CTP in PBS or TGF-β (10 ng/mL for 24 h) induced AMCFs. Scale bar, 50 μm. b, Quantitative assessment of fluorescence intensity from AMCFs. n = 3 biologically independent samples in each group. The results are presented as the mean ± SD. Statistical analysis was performed using one-way ANOVA with the Bonferroni multiple comparison correction. Source data are provided as a Source Data file.

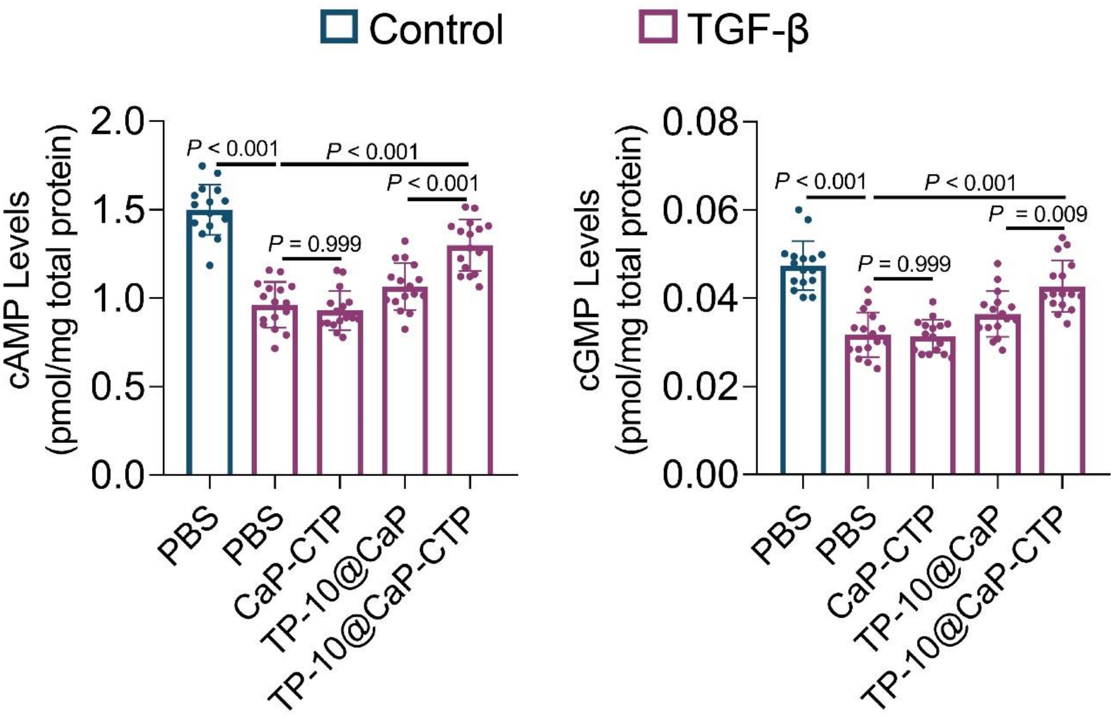

Supplementary Fig. 15. CFs were isolated from adult mice, and serum starved for 24 h, then stimulated with TGF-β (10 ng/mL) or vehicle for another 24 h, prior to treated with 25 μg/mL TP-10@CaP-CTP, TP-10@CaP, CaP-CTP nanoparticles, or vehicle for 1 h. Then, intracellular cAMP and cGMP levels in CFs determined by ELISA assay. n=16 biologically independent samples in each group. The results are presented as the mean ± SD. Statistical analysis was performed using one-way ANOVA with the Bonferroni multiple comparison correction. Source data are provided as a Source Data file.

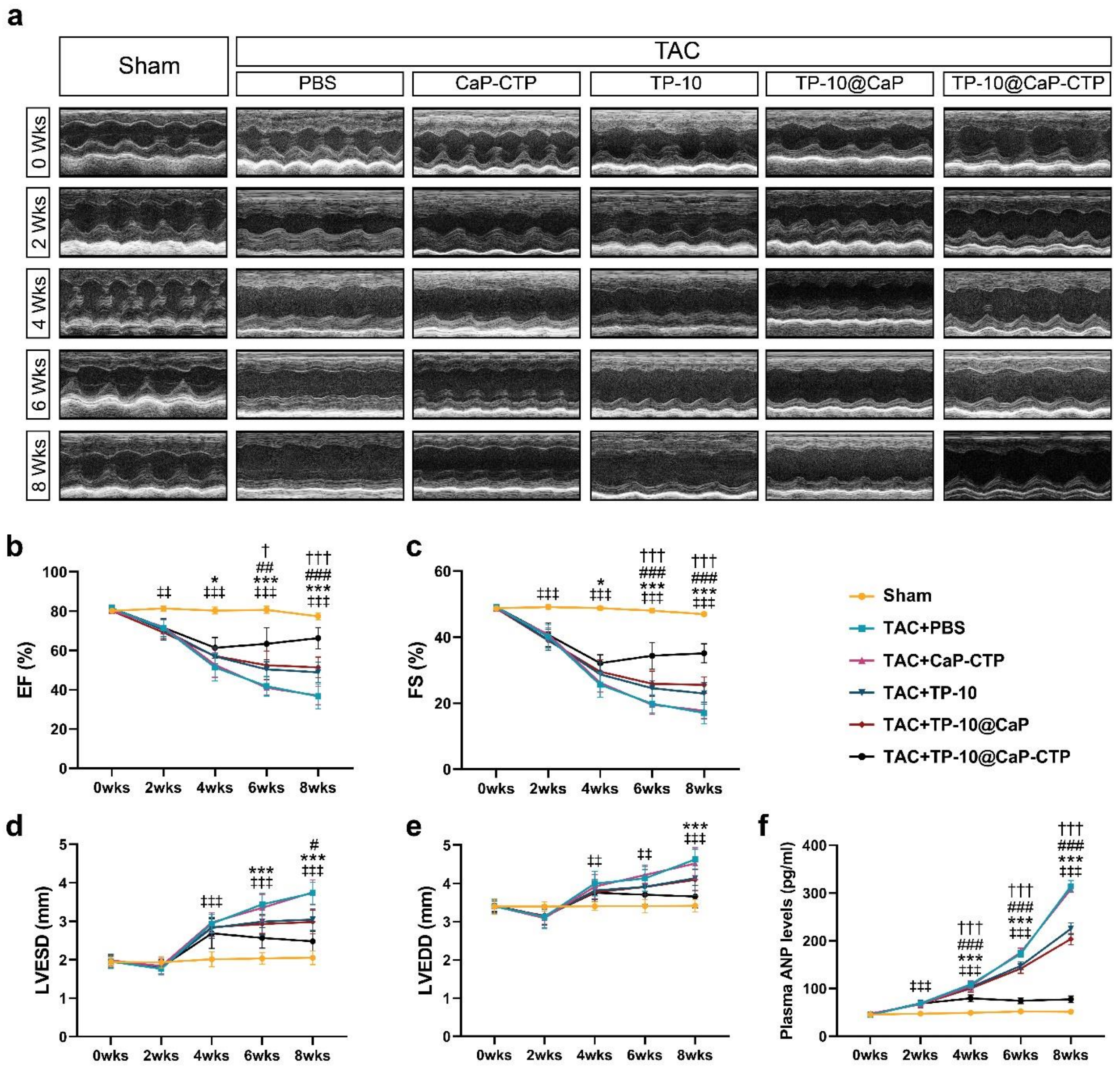

Supplementary Fig. 16. Consecutive echocardiographic and plasma ANP levels monitoring in mice. a, Representative images of echocardiography from each treatment group at 0, 2, 4, 6 and 8 weeks. b-e, the corresponding percentage of ejection fraction (EF%), percentage of fraction shortening (FS%), left ventricular end-systolic diameter (LVESD) and left ventricular end-diastolic diameter (LVEDD) after different treatments. n = 6 mice in each group. f, Quantification of plasma ANP levels at 0, 2, 4, 6 and 8 weeks after different treatments. n = 8 biologically independent samples in each group. The results are presented as the mean ± SD. Statistical analysis was performed using one-way ANOVA with the Bonferroni multiple comparison correction. Source data are provided as a Source Data file.

When TAC+TP-10@CaP-CTP vs TAC+PBS group, *** represents $P < 0.05$, ***, $P < 0.01$, ***, $P < 0.001$.

When TAC+TP-10@CaP-CTP vs TAC+TP-10 group, # represents $P < 0.05$, ##, $P < 0.01$, ###, $P < 0.001$.

When TAC+TP-10@CaP-CTP vs TAC+TP-10@CaP group, † represents $P < 0.05$, ††, $P < 0.01$, †††, $P < 0.001$.

When TAC+PBS vs Sham group, ‡ represents $P < 0.05$, ‡‡, $P < 0.01$, ‡‡‡, $P < 0.001$.

When TAC+PBS vs TAC+ CaP-CTP group, ★ represents $P < 0.05$, ★★, $P < 0.01$, ★★★, $P < 0.001$.

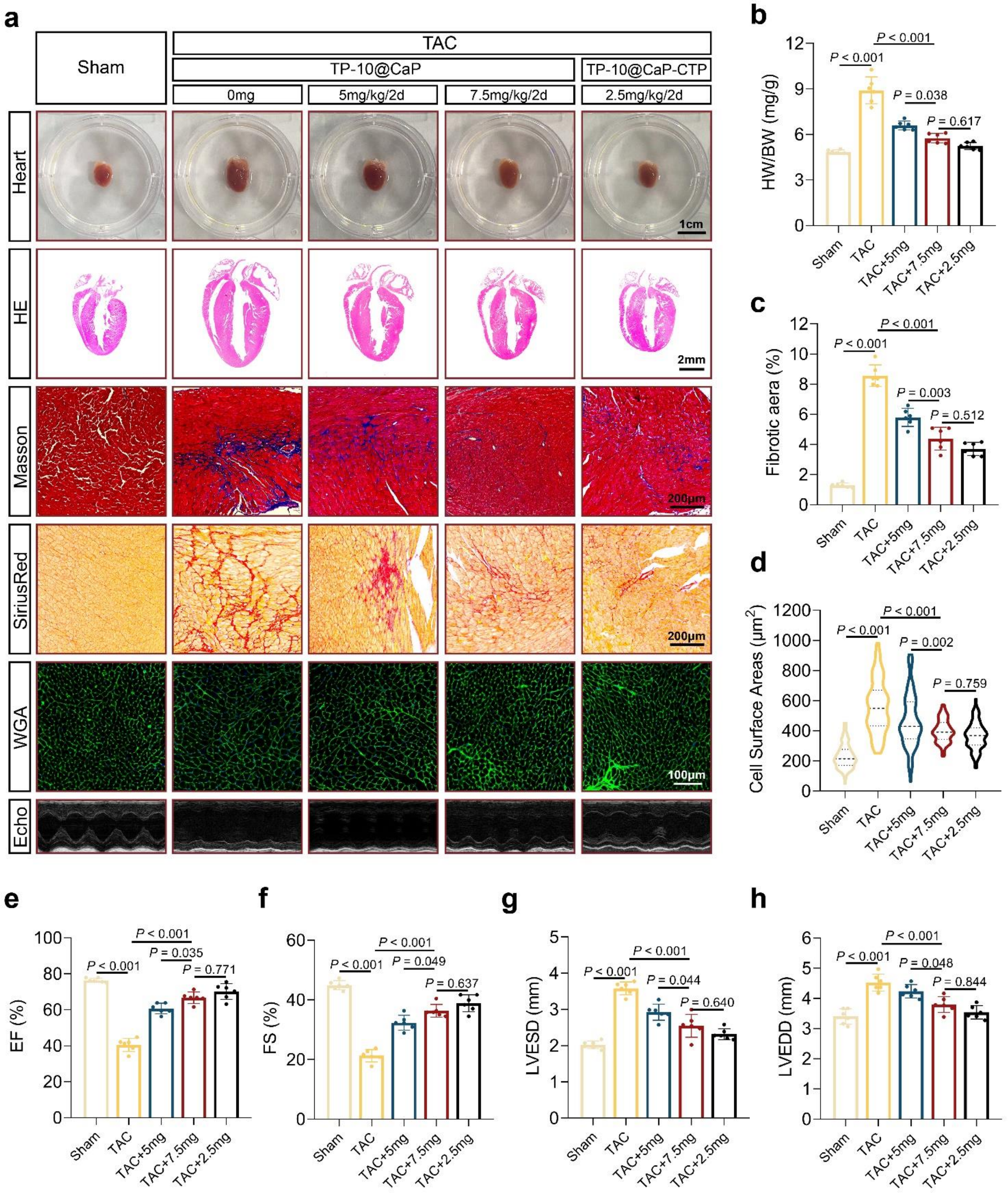

Supplementary Fig. 17. The effect of inhalation treatment with different dosage of TP-10@CaP on prevention of heart failure. Two weeks after TAC- or sham- operation, the mice were treated with different dosages (0 mg/kg/2 days, 5 mg/kg/2 days, or 7.5mg/kg/2 days) of TP-10@CaP or TP-10@CaP-CTP (2.5 mg/kg/2 days) nanoparticles for six weeks via inhalation. a, Representative images of the gross

appearances of whole hearts, heart vertical sections stained with H&E, Masson staining, Sirius red staining, wheat germ agglutinin staining and M-mode echocardiography from mice. b, Ratios of heart weight to body weight (HW/BW). n = 6 mice in each group. c, Statistical analysis of the fibrotic area of the myocardium. n = 6 mice in each group. d, Statistical analysis of the cell surface areas. n = 100-101 CMs from 5 mice in each group. e-h, Percent ejection fraction (EF%), Percent fraction shortening (FS%), left ventricular end-systolic diameter (LVESD), left ventricular end-diastolic diameter (LVEDD). n = 6 mice in each group. The results are presented as the mean ± SD. For b-h, statistical analysis was performed using one-way ANOVA with the Bonferroni multiple comparison correction. Source data are provided as a Source Data file.

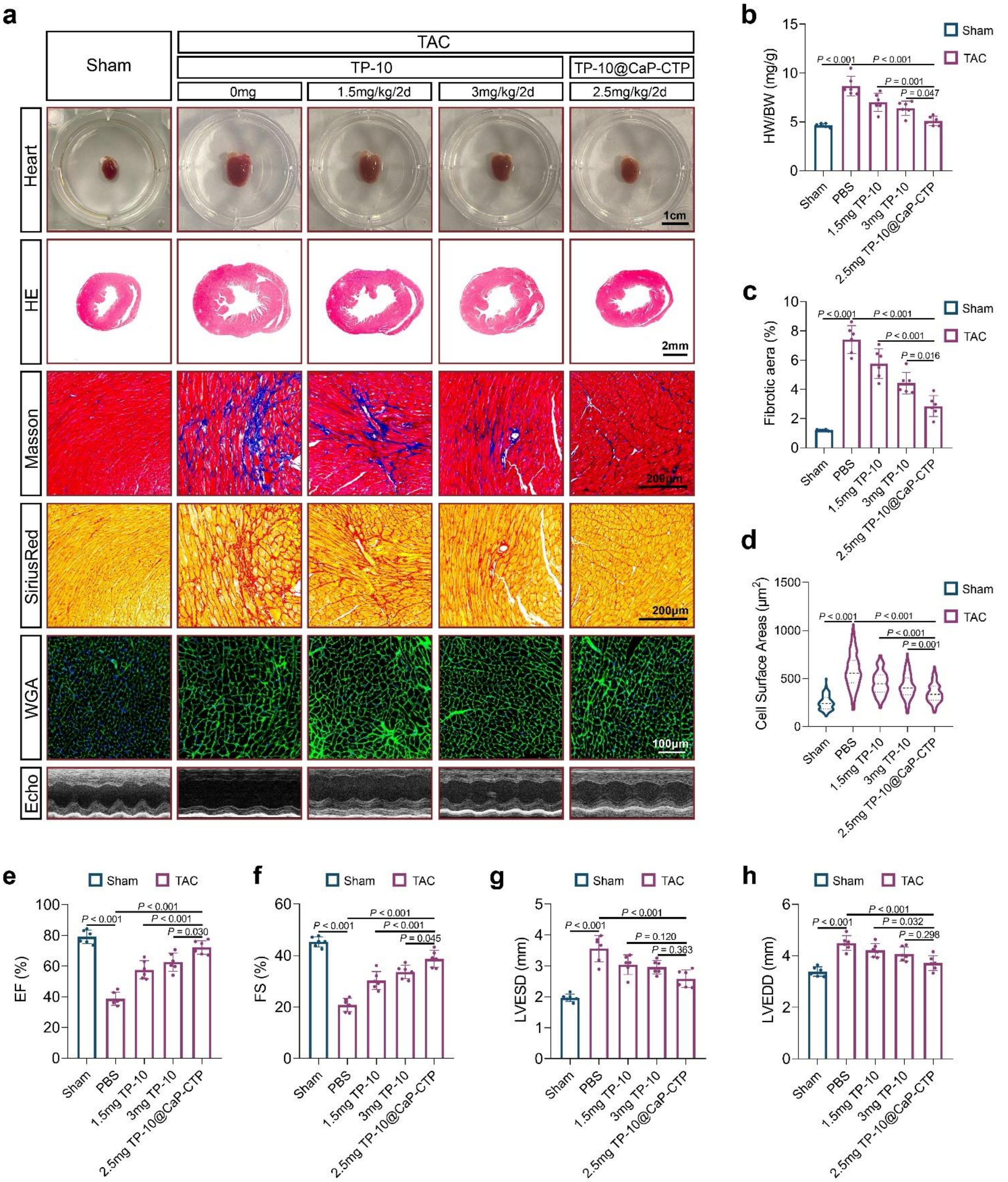

Supplementary Fig. 18. The effect of inhalation treatment with different dosage of free TP-10 on prevention of heart failure. Two weeks after TAC- or sham- operation, the mice were treated with different dosages (1.5 mg/kg/2 days, 3 mg/kg/2 days) of TP-10@CaP, TP-10@CaP-CTP (2.5 mg/kg/2 days) nanoparticles or vehicle for six weeks via inhalation. a, Representative images of the gross

appearances of whole hearts, heart vertical sections stained with H&E, Masson staining, Sirius red staining, wheat germ agglutinin staining and M-mode echocardiography from mice. b, Ratios of heart weight to body weight (HW/BW). n = 6 mice in each group. c, Statistical analysis of the fibrotic area of the myocardium. n = 6 mice in each group. d, Statistical analysis of the cell surface areas. n = 101-105 CMs from 5 mice in each group. e-h, Percent ejection fraction (EF%), Percent fraction shortening (FS%), left ventricular end-systolic diameter (LVESD), left ventricular end-diastolic diameter (LVEDD). n = 6 mice in each group. The results are presented as the mean ± SD. For b-h, statistical analysis was performed using one-way ANOVA with the Bonferroni multiple comparison correction. Source data are provided as a Source Data file.

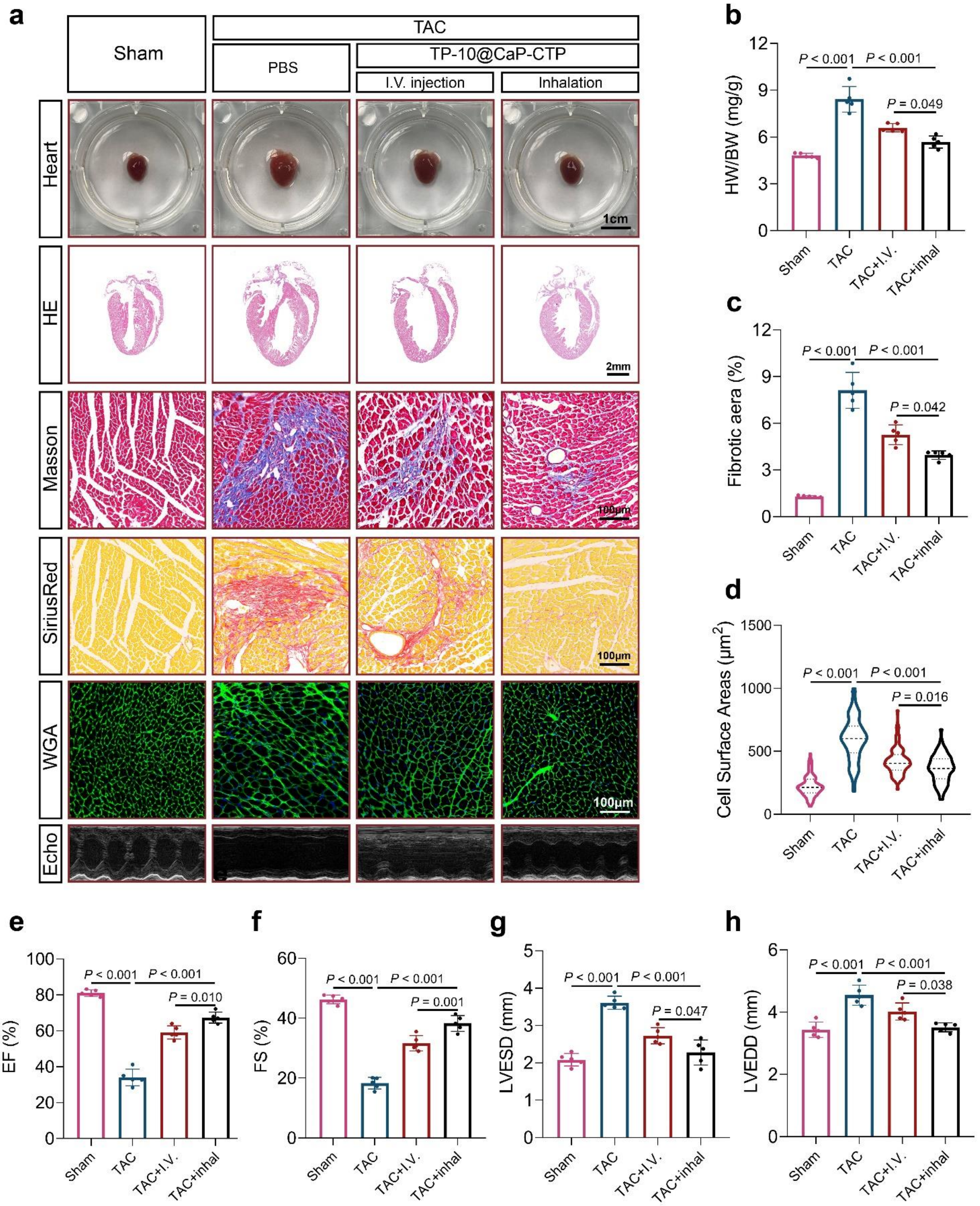

Supplementary Figure 19. Comparison of the effects of I.V. injection and inhalation delivery of TP-10@CaP-CTP on the prevention of heart failure. Two weeks after TAC- or sham- operation, the mice were received inhalation or I.V. treatment of TP-10@CaP-CTP (2.5 mg/kg/2 days) for six weeks. a,

Representative images of the gross appearances of whole hearts, heart vertical-sections with stained H&E, Masson staining, Sirius red staining, wheat germ agglutinin staining and M-mode echocardiography from mice. b, Ratios of heart weight to body weight (HW/BW). n = 5 mice in each group. c, Statistical analysis of the fibrotic area of the myocardium. n = 5 mice in each group. d, Statistical analysis of the cell surface areas n = 72-77 CMs from 5 mice in each group. e-h, Percent ejection fraction (EF%), Percent fraction shortening (FS%), left ventricular end-systolic diameter (LVESD), left ventricular end-diastolic diameter (LVEDD). n = 5 mice in each group. For b-h, statistical analysis was performed using one-way ANOVA with the Bonferroni multiple comparison correction. Source data are provided as a Source Data file.

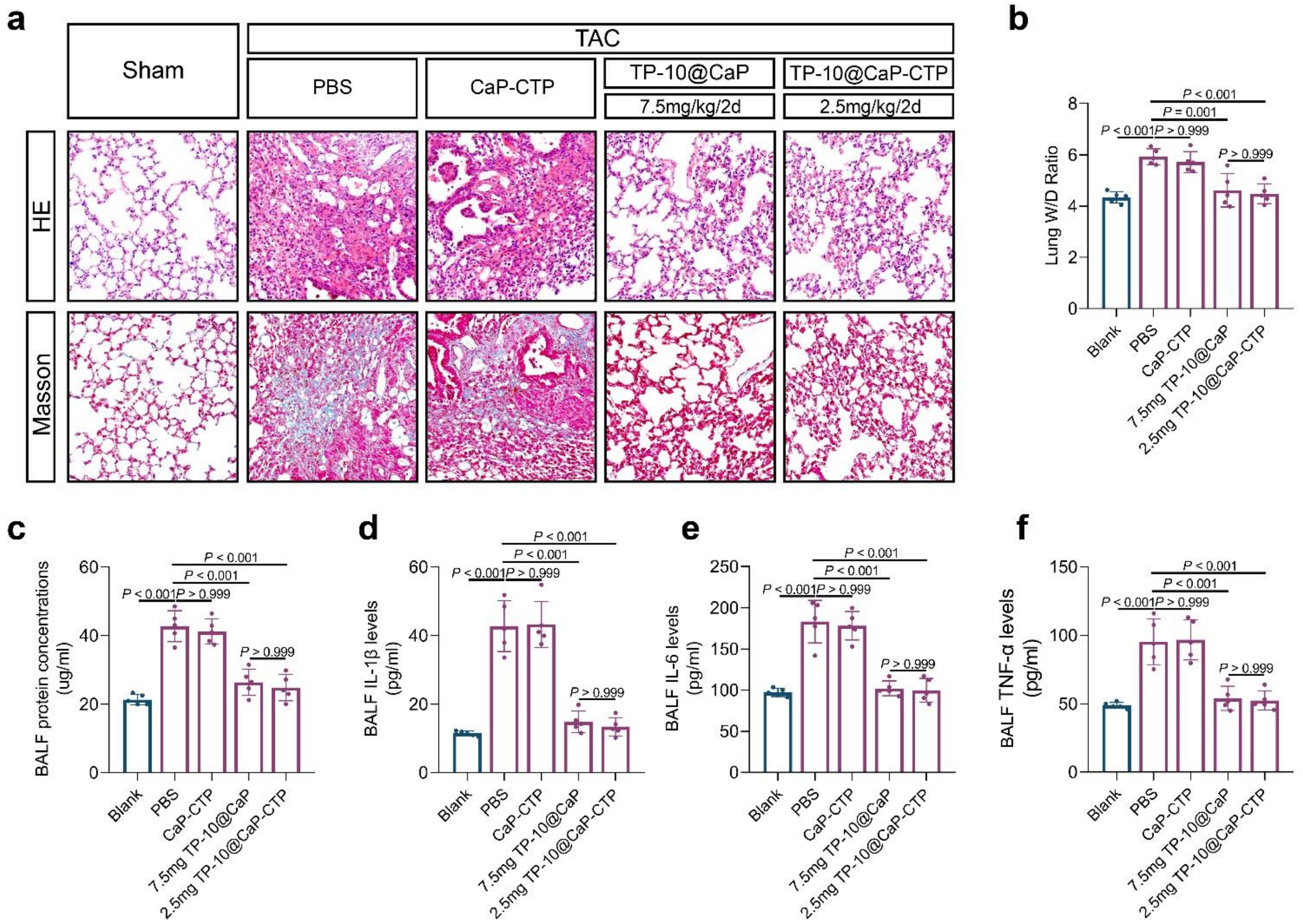

Supplementary Figure 20. Lung safety verification after six weeks of inhalation treatment in TAC mice. a, Representative images of H&E and Masson staining of the lung sections after six weeks of inhalation treatment in TAC mice. n = 6 mice in each group. b, The lung W/D ratio, c, Quantitative assessment of BALF protein concentrations, and d-f, BALF levels of IL-1β, IL-6, and TNF-α. n = 6 biologically independent samples in each group. The results are presented as the mean ± SD. # $P > 0.05$. For b-f, statistical analysis was performed using one-way ANOVA with the Bonferroni multiple comparison correction. Source data are provided as a Source Data file.

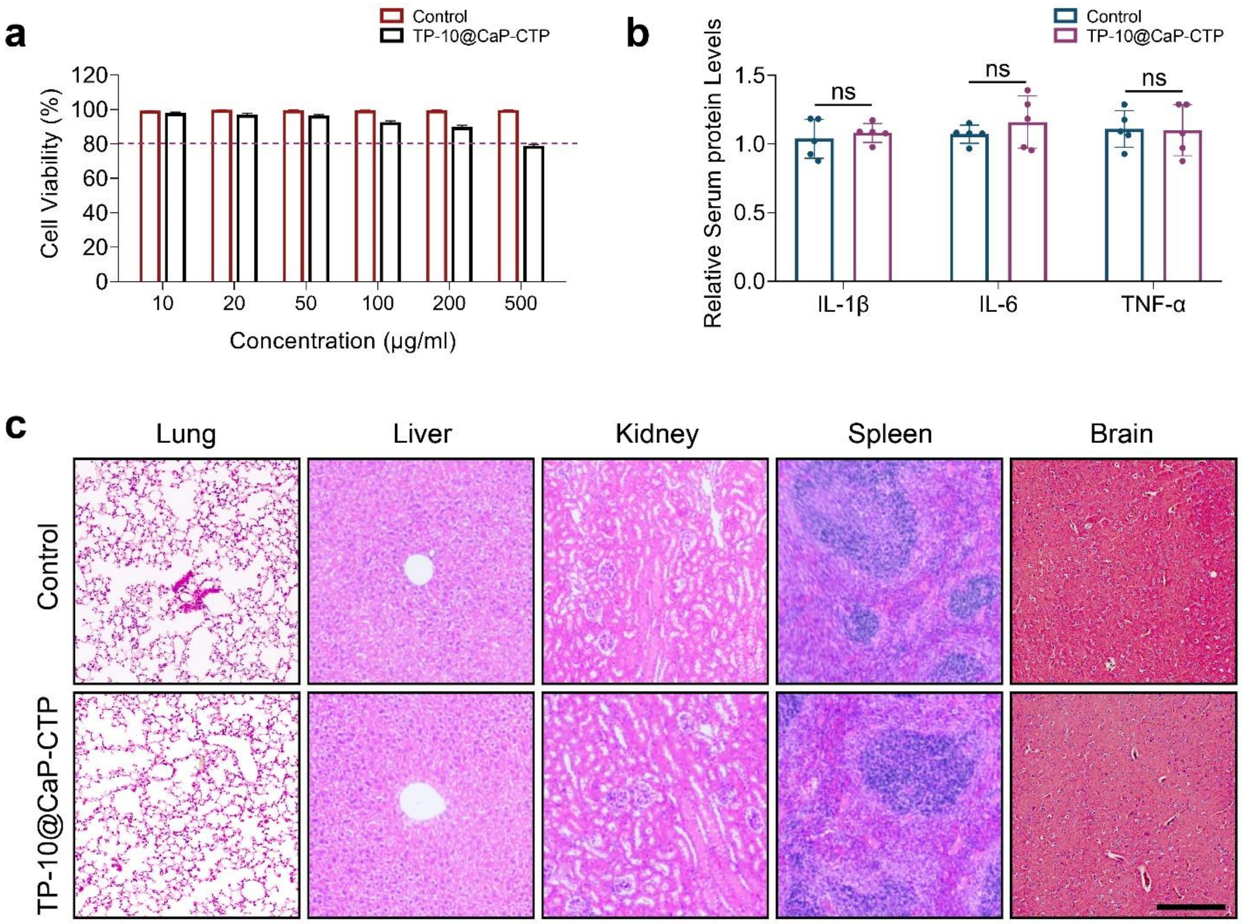

Supplementary Figure 21. Biocompatibility of TP-10@CaP-CTP. a, Cell viability of AC16 cells treated with different concentrations (10, 20, 50, 100, 200, 500 μg/mL) of TP-10@CaP-CTP at 4 h. n = 3 biologically independent samples in each group. b, Quantitative assessment of serum levels of IL-1β, IL-6, and TNF-α from control and inhalation treatment groups at 4 h. n = 5 biologically independent samples in each group. c, Representative images of H&E staining of major organs, including lung, liver, kidney, and spleen. Scale bar, 200 μm. n = 3 biologically independent samples in each group. The results are presented as the mean ± SD. ns $P > 0.05$. statistical analysis between two groups was determined using unpaired two-tailed t tests. Source data are provided as a Source Data file.

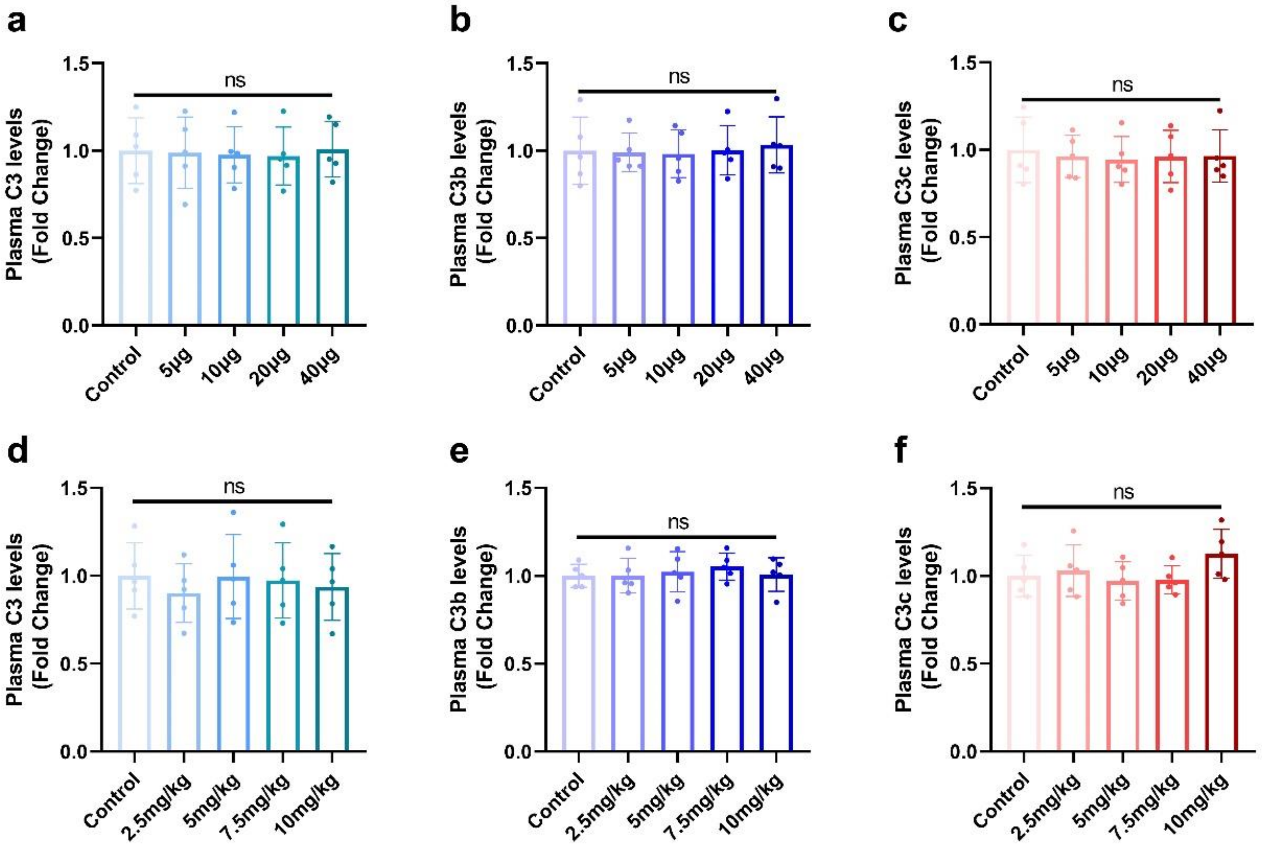

Supplementary Fig. 22. In vitro (a-c) and in vivo (d-e) effect of TP-10@CaP-CTP on complement activation. a-c, for in vitro experiment, 0.5 mL Plasma from mice was incubated for 30 min with various amounts (0, 5, 10, 20 ,40 μg) of TP-10@CaP-CTP and complement activation was monitored by ELISA assay for C3, C3b and C3c levels. n = 5 biologically independent samples in each group. d-f, for in vivo experiment, various amounts (0, 2.5, 5, 7.5, 10 mg/kg) of TP-10@CaP-CTP nanoparticles were administrated via inhalation. Plasma was collected after 1 h and subjected to ELISA assay. n = 5 biologically independent samples in each group. The results are presented as the mean ± SD. Statistical analysis was performed using one-way ANOVA with the Bonferroni multiple comparison correction. Source data are provided as a Source Data file.

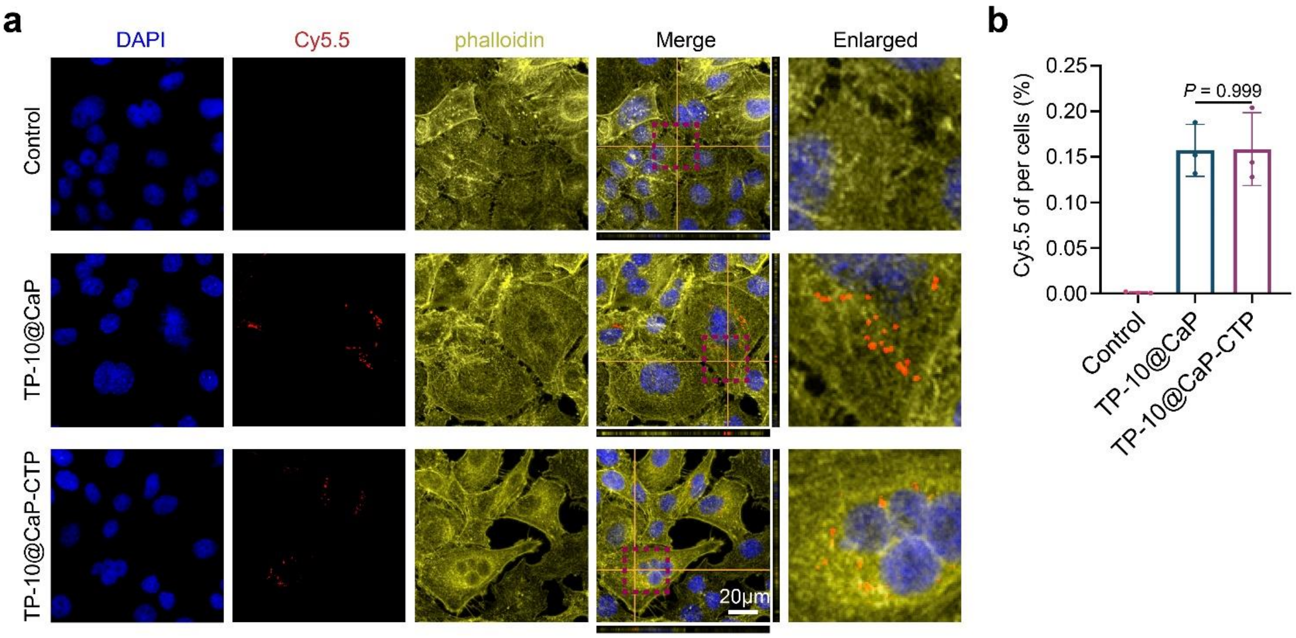

Supplementary Fig. 23. In vitro determination of targeting capacity of TP-10@CaP and TP-10@CaP-CTP in MLE-12. a, Representative images of the intracellular uptak of Cy5.5 labelled TP-10@CaP and TP-10@CaP-CTP in MLE-12. Scale bar, 20 μm. b, Quantitative assessment of fluorescence intensity from MLE-12. n = 3 biologically independent samples in each group. The results are presented as the mean ± SD. Statistical analysis was performed using one-way ANOVA with the Bonferroni multiple comparison correction. Source data are provided as a Source Data file.

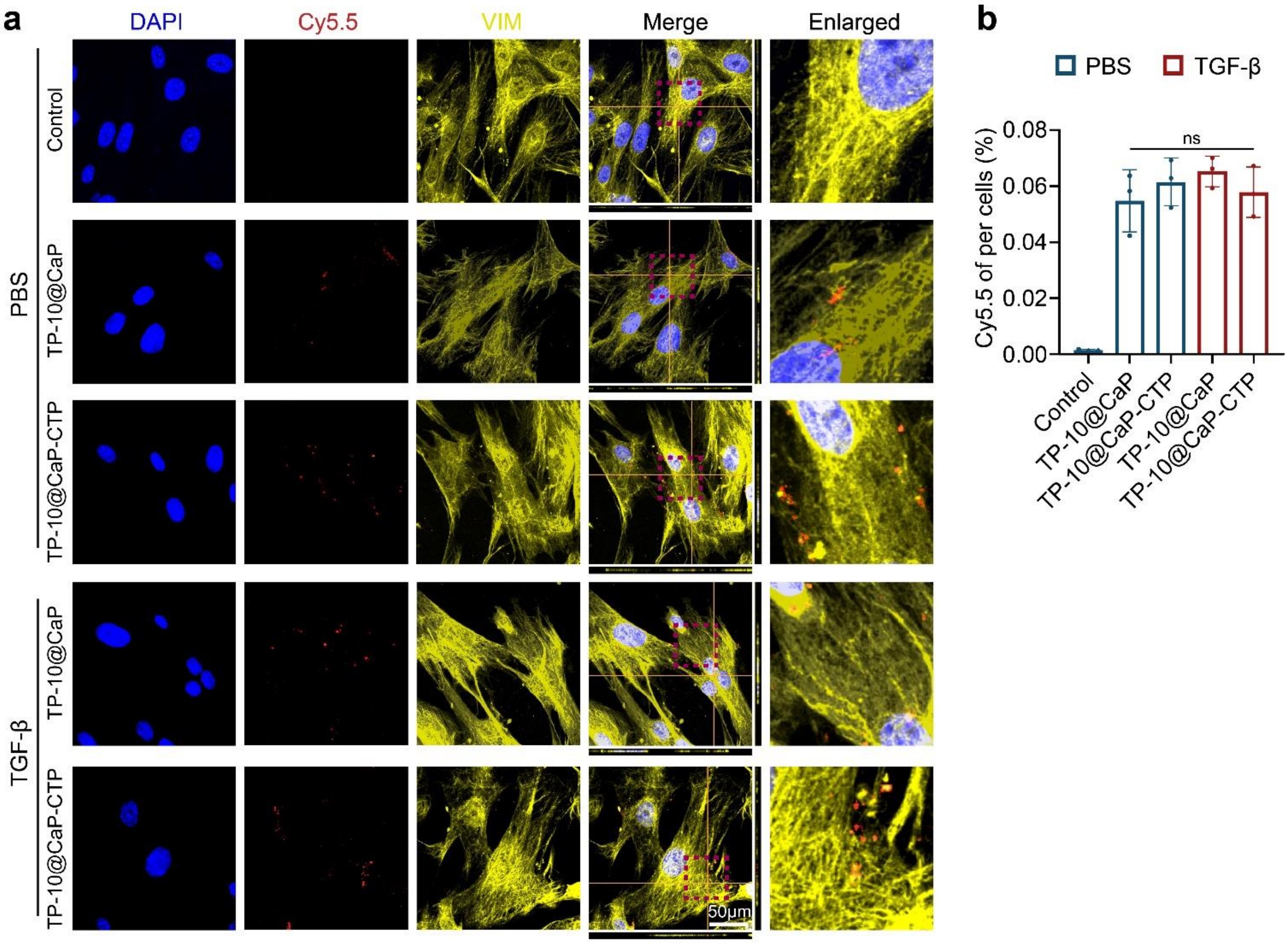

Supplementary Fig. 24. In vitro determination of targeting capacity of TP-10@CaP and TP-10@CaP-CTP in MLFs. a, Representative images of the intracellular uptak of Cy5.5-labelled TP-10@CaP and TP-10@CaP-CTP in PBS or TGF-β induced MLF. Scale bar, 50 μm. b, Quantitative assessment of fluorescence signals from MLF. n = 3 biologically independent samples in each group. The results are presented as the mean ± SD. Statistical analysis was performed using one-way ANOVA with the Bonferroni multiple comparison correction. Source data are provided as a Source Data file.

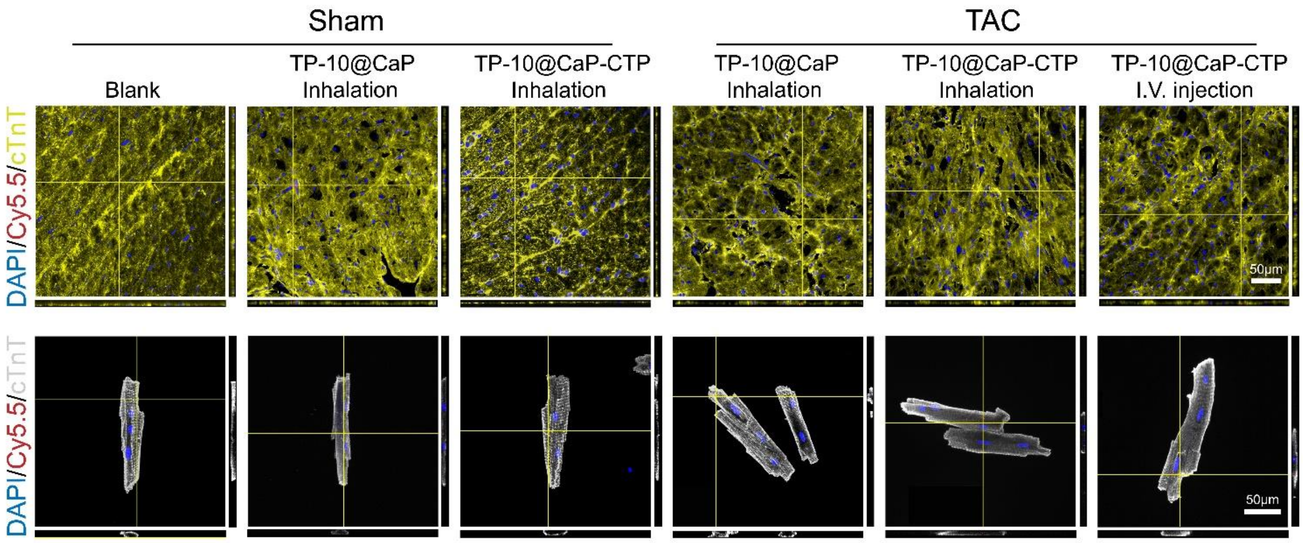

Supplementary Fig. 25. Z-stack analyses from x, y, z view of Fig. 3f (top row) and Fig. 3g (bottom row).

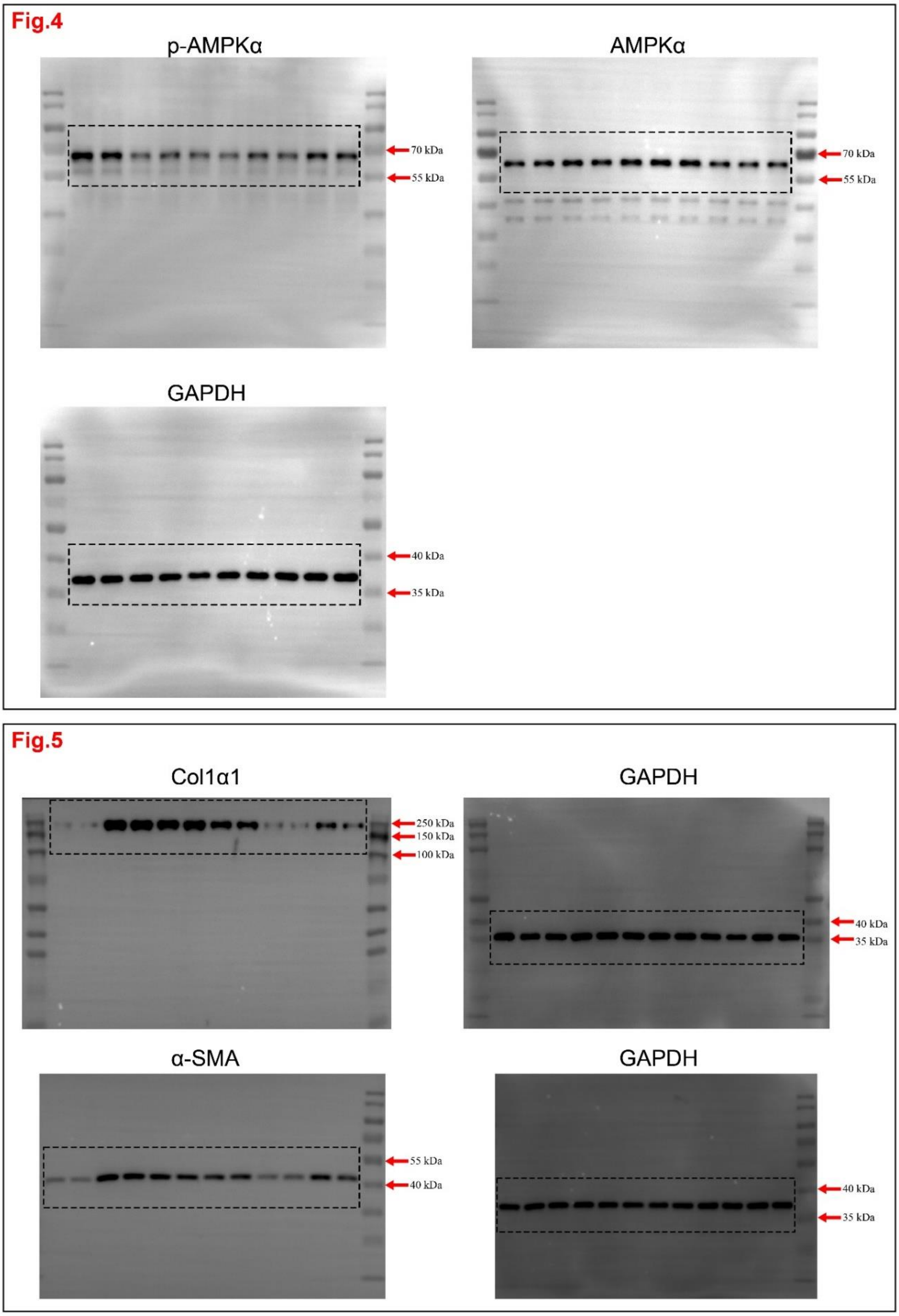

Supplementary Fig. 26. Unprocessed western blot of Fig. 4 and Fig. 5.

Supplementary Tables

Supplementary Table 1. TP-10 level in different organs by HPLC-ESI-MSMS.

	Heart	Liver	Spleen	Lung	Kidney
Blank	ND	ND	ND	ND	ND
TP-10	0.77 ± 0.18	4.08 ± 0.84	0.46 ± 0.21	4.79 ± 0.45	3.25 ± 0.66
TP-10@CaP	3.41 ± 0.68	3.38 ± 0.91	0.40 ± 0.17	6.24 ± 1.03	1.57 ± 0.62
TP-10@CaP-CTP	5.74 ± 1.17	3.07 ± 0.74	0.46 ± 0.13	6.46 ± 1.15	2.27 ± 0.51

Notes: Summary of TP-10 level in different organs by HPLC-ESI-MS/MS from mice received free TP-10, TP-10@CaP or TP-10@CaP-CTP treatment through inhalation approach at 1 h. TP-10 level is expressed as injected dose per gram of tissue (%ID/g). Data are presented as the mean ± SD (n = 6). ND represents not detected.

Supplement Table 2. Biometric and echocardiographic parameters in mice received inhalation treatment.

	Sham	TAC				
Treatment		PBS	CaP-CTP	TP-10	TP-10@CaP	TP-10@CaP-CTP
	n = 8	n = 8	n = 8	n = 8	n = 8	n = 8
HR (beats/min)	465.1 ± 39.9	470.6 ± 23.0	471.8 ± 24.0	461.7 ± 54.0	464.2 ± 36.3	469.0 ± 43.0
EF (%)	79.87 ± 1.81	36.83 ± 3.21	37.66 ± 4.86	55.30 ± 5.732	57.28 ± 6.27	65.68 ± 4.05
FS (%)	45.36 ± 1.02	19.10 ± 2.27	19.93 ± 2.72	29.73 ± 3.39	30.20 ± 4.02	36.20 ± 2.53
LVESD (mm)	2.03 ± 0.12	3.61 ± 0.20	3.59 ± 0.26	3.09 ± 0.28	3.01 + 0.33	2.60 ± 0.23
LVEDD (mm)	3.48 ± 0.14	4.53 ± 0.17	4.51 ± 0.24	4.24 ± 0.31	4.17 ± 0.35	3.74 ± 0.21
E/A (ms^{-1}/ms^{-1})	1.58 ± 0.06	0.92 ± 0.08	0.92 ± 0.10	1.07 ± 0.12	1.11 ± 0.14	1.30 ± 0.08
LVAW, s (mm)	1.19 ± 0.18	1.02 ± 0.09	1.04 ± 0.12	1.27 ± 0.17	1.30 ± 0.21	1.22 ± 0.15
LVAW, d (mm)	0.79 ± 0.03	0.65 ± 0.09	0.67 ± 0.06	0.86 ± 0.09	0.89 ± 0.08	0.82 ± 0.08
LVPW, s (mm)	1.08 ± 0.07	0.98 ± 0.13	1.00 ± 0.14	1.13 ± 0.09	1.16 ± 0.13	1.09 ± 0.13
LVPW, d (mm)	0.71 ± 0.08	0.61 ± 0.05	0.63 ± 0.06	0.77 ± 0.09	0.79 ± 0.11	0.73 ± 0.09
CO (mL/min)	19.34 ± 2.09	13.44 ± 1.32	13.66 ± 1.73	16.45 ± 1.88	17.75 ± 2.12	18.45 ± 1.72
SV (μL)	40.37 ± 2.11	27.44 ± 3.59	26.45 ± 3.04	32.50 ± 4.04	33.37 ± 2.80	36.25 ± 1.55
HW/BW (mg/g)	4.76 ± 0.23	8.23 ± 0.97	8.20 ± 1.29	6.99 ± 0.35	6.86 ± 0.29	5.57 ± 0.34
AVPP (mmHg)	1.63 ± 0.15	37.15 ± 2.68	36.14 ± 1.91	36.64 ± 2.19	36.39 ± 2.71	37.36 ± 2.23

Notes: HR: heart rate, EF: ejection fraction, FS: fraction shortening, LVESD: left ventricular end-systolic diameter, LVEDD: left ventricular end-diastolic diameter, LVAW： left ventricular anterior wall diameter, LVPW: left ventricular posterior wall diameter, CO: cardiac output, SV: stroke volume, HW: heart weight, BW: body weight, AVPP: Aortic velocity peak pressure. Statistical analysis was performed using one-way ANOVA with Bonferroni multiple-comparison correction. Data are presented as the mean ± SD.

Supplement Table 3. Biometric and echocardiographic parameters in mice received TP-10@CaP and TP-10@CaP-CTP inhalation treatment

Treatment	Sham	TAC			
		0mg/kg TP-10@CaP	5mg/kg TP-10@CaP	7.5mg/kg TP-10@CaP	2.5mg/kg TP-10@CaP-CTP
	n = 6	n = 6	n = 6	n = 6	n = 6
HR (beats/min)	465.0 ± 15.9	471.1 ± 13.2	462.0 ± 18.0	466.5 ± 18.1	469.0 ± 15.0
EF (%)	76.46 ± 1.21	40.53 ± 3.62	60.73 ± 2.95	67.77 ± 3.14	68.22 ± 4.41
FS (%)	44.93 ± 1.53	21.30 ± 2.08	32.38 ± 2.49	37.39 ± 2.12	37.91 ± 2.81
LVESD (mm)	2.02 ± 0.11	3.58 ± 0.18	2.93 ± 0.23	2.45 ± 0.32	2.42 ± 0.15
LVEDD (mm)	3.40 ± 0.25	4.53 ± 0.28	4.14 ± 0.22	3.70 ± 0.26	3.69 ± 0.22
LVAW, s (mm)	1.21 ± 0.13	0.99 ± 0.07	1.29 ± 0.18	1.22± 0.14	1.24 ± 0.13
LVAW, d (mm)	0.77 ± 0.09	0.70 ± 0.07	0.85 ± 0.09	0.82 ± 0.11	0.83 ± 0.11
LVPW, s (mm)	1.11 ± 0.05	0.95 ± 0.03	1.17 ± 0.15	1.16 ± 0.11	1.13 ± 0.11
LVPW, d (mm)	0.69 ± 0.06	0.58 ± 0.05	0.75 ± 0.08	0.72 ± 0.07	0.71 ± 0.08
CO (mL/min)	21.27 ± 1.74	12.95 ± 1.21	17.47 ± 2.00	19.35 ± 1.50	20.13 ± 1.76
SV (μL)	41.47 ± 2.25	26.58 ± 1.57	35.94 ± 2.82	39.34± 1.67	39.94 ± 2.35
HW/BW (mg/g)	4.86 ± 0.13	8.91 ± 0.88	6.60 ± 0.29	5.76 ± 0.32	5.85 ± 0.23
AVPP (mmHg)	1.68 ± 0.20	36.88 ± 2.26	37.04 ± 2.67	37.01 ± 2.58	37.10 ± 2.66

Notes: HR: heart rate, EF: ejection fraction, FS: fraction shortening, LVESD: left ventricular end-systolic diameter, LVEDD: left ventricular end-diastolic diameter, LVAW： left ventricular anterior wall diameter, LVPW: left ventricular posterior wall diameter, CO: cardiac output, SV: stroke volume, HW: heart weight, BW: body weight, AVPP: Aortic velocity peak pressure. Statistical analysis was performed using one-way ANOVA with Bonferroni multiple-comparison correction. Data are presented as the mean ± SD.

Supplementary Table 4. Biometric and echocardiographic parameters in mice received 50 µL TP-10@CaP-CTP inhalation or I.V. treatment

	Sham	TAC		
Treatment		PBS	TP-10@CaP-CTP (I.V)	TP-10@CaP (inhalation)
	n = 5	n = 5	n = 5	n = 5
HR (beats/min)	451.3 ± 19.1	446.0 ± 19.2	456.3 ± 24.0	448.4 ± 18.0
EF (%)	81.04 ± 1.82	34.04 ± 4.67	59.11 ± 3.69	67.37 ± 3.09
FS (%)	46.30 ± 1.49	18.31 ± 1.95	31.69 ± 2.55	38.26 ± 2.61
LVESD (mm)	2.09 ± 0.17	3.61 ± 0.17	2.73 ± 0.22	2.28 ± 0.34
LVEDD (mm)	3.44 ± 0.25	4.55 ± 0.32	4.02 ± 0.38	3.51 ± 0.15
LVAW, s (mm)	1.16 ± 0.07	1.04 ± 0.03	1.28 ± 0.11	1.21 ± 0.11
LVAW, d (mm)	0.82 ± 0.07	0.68 ± 0.05	0.86 ± 0.07	0.84 ± 0.05
LVPW, s (mm)	1.15 ± 0.05	0.92 ± 0.07	1.24 ± 0.08	1.19 ± 0.08
LVPW, d (mm)	0.72 ± 0.01	0.63 ± 0.04	0.81 ± 0.04	0.77 ± 0.04
CO (mL/min)	20.54 ± 2.21	13.84 ± 0.71	17.33 ± 0.73	19.56 ± 1.28
SV (μL)	39.33 ± 2.58	27.41 ± 1.97	36.82 ± 1.87	38.22 ± 1.42
HW/BW (mg/g)	4.82 ± 0.15	8.43 ± 0.82	6.60 ± 0.27	5.68 ± 0.39
AVPP (mmHg)	1.74 ± 0.21	39.49 ± 3.93	38.51 ± 3.57	39.19 ± 3.50

Notes: HR: heart rate, EF: ejection fraction, FS: fraction shortening, LVESD: left ventricular end-systolic diameter, LVEDD: left ventricular end-diastolic diameter, LVAW: left ventricular anterior wall diameter, LVPW: left ventricular posterior wall diameter, CO: cardiac output, SV: stroke volume, HW: heart weight, BW: body weight, AVPP: Aortic velocity peak pressure. Statistical analysis was performed using one-way ANOVA with Bonferroni multiple-comparison correction. Data are presented as the mean ± SD.

Supplement Table 5. Primer sequences used for real-time qPCR analysis in this study

Gene	Species	Sequence (Forward)	Sequence (Reverse)
Nppa	Mouse	TACAGTGCGGTGTCCAACACAG	TGCTTCCTCAGTCTGCTCACTC
Nppb	Mouse	TCCTAGCCAGTCTCCAGAGCAA	GGTCCTTCAAGAGCTGTCTCTG
Myh7	Mouse	ACTGTCAACACTAAGAGGGTCA	TTGGATGATTTGATTTCCAGGG
Col1a1	Mouse	CCTCAGGGTATTGCTGGACAAC	CAGAAGGACCTTGTTTGCCAGG
Col3a1	Mouse	GACCAAAAGGTGATGCTGGACAG	CAAGACCTCGTGCTCCAGTTAG
Acta1	Mouse	GCTTCGCTGGTGATGATGCTC	AGTTGGTGATGATGCCGTGTTC
Fn1	Mouse	GCTCAGCAAATCGTGCAGC	CTAGGTAGGTCCGTTCCCACT
Gapdh	Mouse	TCAAGAAGGTGGTGAAGCAG	TGGGAGTTGCTGTTGAAGTC
