# Supplementary material for: Inhalable cardiac targeting peptide modified nanomedicine prevents pressure overload heart failure in male mice
Source: Nat Commun. 2024 Jul 18;15:6058. doi: 10.1038/s41467-024-50312-1 (PMC11258261; doi:10.1038/s41467-024-50312-1)
Supplement: Supplementary file 2 — Reporting Summary [file 41467_2024_50312_MOESM2_ESM.pdf]

nature portfolio

Corresponding author(s): Xianhong Shu, Yuanyi Zheng, Jianrong Wu

Last updated by author(s): Dec 10, 2023

Reporting Summary

Nature Portfolio wishes to improve the reproducibility of the work that we publish. This form provides structure for consistency and transparency in reporting. For further information on Nature Portfolio policies, see our Editorial Policies and the Editorial Policy Checklist.

Statistics

For all statistical analyses, confirm that the following items are present in the figure legend, table legend, main text, or Methods section.

n/a	Confirmed	
☐	☒	The exact sample size (*n*) for each experimental group/condition, given as a discrete number and unit of measurement
☐	☒	A statement on whether measurements were taken from distinct samples or whether the same sample was measured repeatedly
☐	☒	The statistical test(s) used AND whether they are one- or two-sided<br>*Only common tests should be described solely by name; describe more complex techniques in the Methods section.*
☒	☐	A description of all covariates tested
☐	☒	A description of any assumptions or corrections, such as tests of normality and adjustment for multiple comparisons
☐	☒	A full description of the statistical parameters including central tendency (e.g. means) or other basic estimates (e.g. regression coefficient) AND variation (e.g. standard deviation) or associated estimates of uncertainty (e.g. confidence intervals)
☐	☒	For null hypothesis testing, the test statistic (e.g. F, t, r) with confidence intervals, effect sizes, degrees of freedom and P value noted<br>*Give P values as exact values whenever suitable.*
☒	☐	For Bayesian analysis, information on the choice of priors and Markov chain Monte Carlo settings
☒	☐	For hierarchical and complex designs, identification of the appropriate level for tests and full reporting of outcomes
☒	☐	Estimates of effect sizes (e.g. Cohen's d, Pearson's r), indicating how they were calculated

Our web collection on statistics for biologists contains articles on many of the points above.

Software and code

Policy information about availability of computer code

Data collection

1. qPCR: Bio-Rad’s CFX96
2. Western Blot: Tanon 5200
3. Immunofluorescence: Olympus FV3000
4. In vivo imaging system: VISQUE
5. Mice echo: Vevo 3100
6. Transmission electron microscopy (TEM) images: JEM-2100F field emission TEM (JEOL, Tokyo, Japan)
7. The hydrodynamic particle size and the corresponding zeta potential: Nano ZS90 Zetasizer Nanoseries (Malvern, UK).
8. FT-IR spectra: IR Prestige-21 spectrometer (Shimadzu, Japan)
9. X-ray photo-electron spectra (XPS): EscaLab 250Xi electron spectrometer (Thermo Fisher Scientific, USA)
10. Powder X-ray diffraction (XRD): D8 ADVANCE X-ray diffractometer (Bruker, Billerica, MA, USA).
11. HPLC-ESI-MS/MS: EXPEC 5250, Agilent Technologies, Santa Clara, US

Data analysis

Statistics analysis were performed with Microsoft Excel 2018, Image J 1.52 and GraphPad Prism 8.0 software (San Diego, CA, USA).

For manuscripts utilizing custom algorithms or software that are central to the research but not yet described in published literature, software must be made available to editors and reviewers. We strongly encourage code deposition in a community repository (e.g. GitHub). See the Nature Portfolio guidelines for submitting code & software for further information.

Data

Policy information about availability of data

All manuscripts must include a data availability statement. This statement should provide the following information, where applicable:
- Accession codes, unique identifiers, or web links for publicly available datasets
- A description of any restrictions on data availability
- For clinical datasets or third party data, please ensure that the statement adheres to our policy

The data supporting the findings in this study are available within the manuscript,its Supplementary Information file and the Source Data file.Source data are provided with this paper.

Research involving human participants, their data, or biological material

Policy information about studies with human participants or human data. See also policy information about sex, gender (identity/presentation), and sexual orientation and race, ethnicity and racism.

Reporting on sex and gender	Not applicable.
Reporting on race, ethnicity, or other socially relevant groupings	Not applicable.
Population characteristics	Not applicable.
Recruitment	Not applicable.
Ethics oversight	Not applicable.

Note that full information on the approval of the study protocol must also be provided in the manuscript.

Field-specific reporting

Please select the one below that is the best fit for your research. If you are not sure, read the appropriate sections before making your selection.

☒ Life sciences ☐ Behavioural & social sciences ☐ Ecological, evolutionary & environmental sciences

For a reference copy of the document with all sections, see nature.com/documents/nr-reporting-summary-flat.pdf

Life sciences study design

All studies must disclose on these points even when the disclosure is negative.

Sample size	No statistical methods were used to predetermine the sample sizes.The group sizes were determined based on our experimental experience, which are indicated in each figure and were enough to facilitate the statistical analysis. For in vivo studies, sample sizes were chosen based on the minimum number of animals/replicates according to the previously published articles to support meaningful conclusions.
Data exclusions	No data were excluded from the analysis.
Replication	All attempts to replicate results were successful. in in vitro studies, experiments were biologically replicated three times and obtained similar results. For in vivo experiments, we report pooled results from multiple experiments with similar results or the data shown correspond to one representative experiment of at least 3 biological replicates.
Randomization	Mice were randomly selected, and no algorithm was used.
Blinding	All the data collection and analysis were from blinded with randomized samples.

Reporting for specific materials, systems and methods

We require information from authors about some types of materials, experimental systems and methods used in many studies. Here, indicate whether each material, system or method listed is relevant to your study. If you are not sure if a list item applies to your research, read the appropriate section before selecting a response.

Materials & experimental systems

n/a	Involved in the study	
☐	☒	Antibodies
☐	☒	Eukaryotic cell lines
☒	☐	Palaeontology and archaeology
☐	☒	Animals and other organisms
☒	☐	Clinical data
☒	☐	Dual use research of concern
☒	☐	Plants

Methods

n/a	Involved in the study	
☒	☐	ChIP-seq
☒	☐	Flow cytometry
☒	☐	MRI-based neuroimaging

Antibodies

Antibodies used

Primary antibodies for western blot analysis: Anti-AMPKα (#2532, 1: 1000, Cell Signaling Technology, MA, USA), Anti-phospho-AMPKα (#2535, 1: 1000, Cell Signaling Technology, MA, USA), Anti-α-smooth muscle actin (#19245T, 1: 1000, Cell Signaling Technology, MA, USA), Anti-antibodies against Col1α1 (#ab270993, 1:1000, Abcam, Cambridge, UK).

Primary antibodies for immunofluorescence: Anti-cardiac troponin T (#ab209813, 1: 3000 for cardiomyocytes, Abcam, Cambridge, UK), Anti-Col1α1 (#ab270993, 1:2000, Abcam, Cambridge, UK), Anti-Vimentin (#ab24525, 1:300, Abcam, Cambridge, UK) , Anti-cardiac troponin T (#ab8295, 1: 2000 for myocardial tissue, Abcam, Cambridge, UK), Anti-α-smooth muscle actin (#19245T, 1:500, Cell Signaling Technology, MA, USA), Anti-Discoidin domain receptor 2 (#sc-81707, 1:50, Santa Cruz Biotechnology, Dallas, USA), Anti-CD31 (#DIA-310, 1:200, BIOZOL, Eching, Germany), Anti-Phalloidin (#40736ES75, 1:1000, Yeasen, Shanghai, China)

Validation

Antibodies have been validated by company and detailed information could be found on the manufactures' websites.

Anti-AMPKα (#2532, Cell Signaling Technology, MA, USA)
https://www.cellsignal.cn/products/primary-antibodies/ampka-antibody/2532

Anti-phospho-AMPKα (#2535, Cell Signaling Technology, MA, USA)
https://www.cellsignal.cn/products/primary-antibodies/phospho-ampka-thr172-40h9-rabbit-mab/2535

Anti-α-smooth muscle actin (#19245T, Cell Signaling Technology, MA, USA)
https://www.cellsignal.cn/products/primary-antibodies/a-smooth-muscle-actin-d4k9n-xp-rabbit-mab/19245

Anti-Col1α1 (#ab270993, Abcam, Cambridge, UK)
https://www.abcam.cn/products/primary-antibodies/collagen-i-antibody-epr24331-53-ab270993.html

Anti-cardiac troponin T (#ab209813, Abcam, Cambridge, UK)
https://www.abcam.cn/products/primary-antibodies/cardiac-troponin-t-antibody-epr20266-ab209813.html

Anti-Vimentin (#ab24525, Abcam, Cambridge, UK)
https://www.abcam.cn/products/primary-antibodies/vimentin-antibody-ab24525.html

Anti-cardiac troponin T (#ab8295, Abcam, Cambridge, UK)
https://www.abcam.cn/products/primary-antibodies/cardiac-troponin-t-antibody-1c11-ab8295.html

Anti-Discoidin domain receptor 2 (#sc-81707, Santa Cruz Biotechnology, Dallas, USA)
https://www.scbt.com/p/ddr2-antibody-3b11e4

Anti-Phalloidin (#40736ES75, Yeasen, Shanghai, China)
https://www.yeasen.com/products/detail/340

Eukaryotic cell lines

Policy information about cell lines and Sex and Gender in Research

Cell line source(s)	AC16 (Chinese Academy of Science Cell Bank), MLE-12 (Shanghai Zhong Qiao Xin Zhou Biotechnology Co.,Ltd.)
Authentication	Cell lines were authenticated by STR profiling.
Mycoplasma contamination	All the cell lines were tested termly and they were negative for mycoplasma contamination.
Commonly misidentified lines (See ICLAC register)	No commonly misidentified cell lines were used.

Animals and other research organisms

Policy information about studies involving animals; ARRIVE guidelines recommended for reporting animal research, and Sex and Gender in Research

Laboratory animals	Male C57BL/6J mice (8-week-old) were obtained from Vital River Laboratory Animal Technology Co., Ltd. (Beijing, China). 1- to 3-day-old male Sprague-Dawley rats were obtained from Vital River Laboratory Animal Technology Co., Ltd. (Beijing, China). The mice were euthanized in a CO2 chamber for experiments.
Wild animals	No wild animals were used in the study.
Reporting on sex	Only male mice were used in this study. To the best of our knowledge, there is no evidence suggests that sex differences in mice affect the efficiency of lung-based drug delivery. Besides, estrogen has a protective effect on heart failure and may influence the results of animal experiments. Therefore, we only used male mice for animal studies. Both sexes of rats were used to isolate neonatal ventricular cardiomyocytes since it is difficult to separate male and female in neonates.
Field-collected samples	No field collected samples were used in the study.
Ethics oversight	All the animal experiments were performed according to the guidelines for the Care and Use of Laboratory Animals published by the National Research Council (U.S.) Institute for Laboratory Animal Research and were approved by the Ethics Committee of Zhongshan Hospital, Fudan University, Shanghai, China (approval No. 2023-004).

Note that full information on the approval of the study protocol must also be provided in the manuscript.
